# Coupling dynamic catalyst regeneration with convective transport enables stable and selective glycerol electrooxidation on platinum

**DOI:** 10.1039/d6ey00090h

**Published:** 2026-08-19

**Authors:** Kanyapat Plub-in, Hanzhi Ye, Carlos G. Morales-Guio, Maria Magdalena Titirici, Ifan E.L. Stephens, Anna Hankin

**Affiliations:** a Department of Chemical Engineering, Imperial College London London SW7 2AZ UK anna.hankin@imperial.ac.uk; b Department of Chemical and Biomolecular Engineering, University of California, Los Angeles California CA 90095 USA; c Advanced Institute for Materials Research (AIMR), Tohoku University 2-1-1 Katahira, Aoba-ku Sendai 980-8577 Japan; d Department of Materials, Imperial College London London SW7 2AZ UK

## Abstract

Electrochemical glycerol oxidation on platinum is governed by surface poisoning, competing faradaic and non-faradaic pathways, and mass-transport effects, complicating mechanistic understanding and catalyst optimization. Here, we combine controlled hydrodynamics with dynamic potential modulation to enhance glycerol electrooxidation without complex catalyst design. Using a rotating polycrystalline platinum cylinder electrode in alkaline electrolyte, we develop a three-step pulsed potential protocol comprising glycerol oxidation, oxidative removal of poisoning intermediates, and surface regeneration *via* oxide reduction. The optimized protocol sustains high activity and stability, delivering current densities up to 12–14 mA cm^−2^ and increasing glycerate faradaic efficiency to ∼40%. Mild rotation maximises activity, while higher rotation rates enhance glyceraldehyde removal and its non-faradaic conversion to lactate, increasing lactate faradaic efficiency from 2% to 20%. These findings demonstrate that synergistic control of catalyst regeneration and hydrodynamics provides a powerful strategy to tune activity and selectivity in glycerol electrooxidation.

## Abstract 2

Improving glycerol-to-lactic acid selectivity through three-step pulsed potential operation coupled with convective transport control.

## Graphical abstract

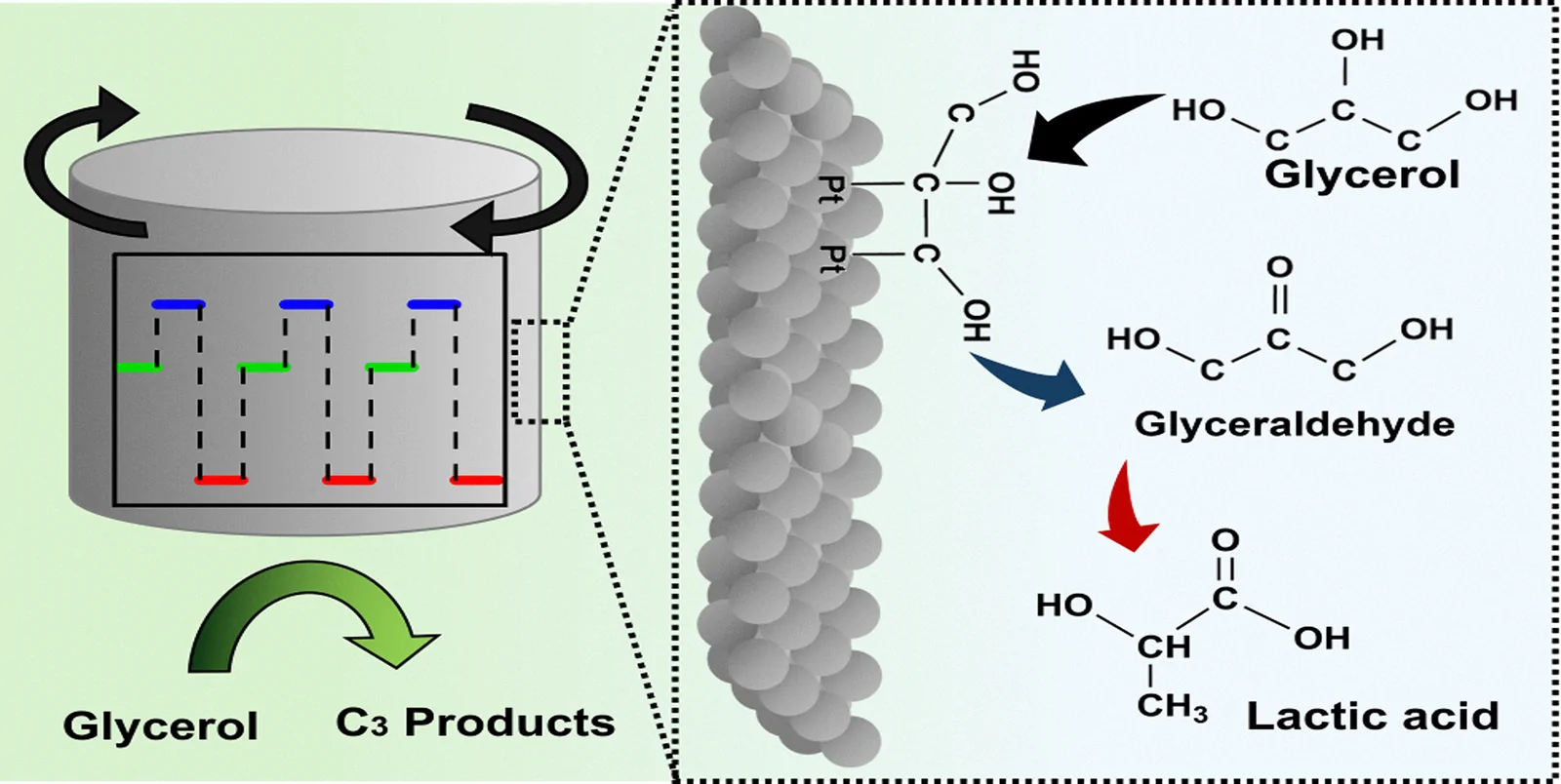

**Broader context**

The transition to circular and low-carbon manufacturing has increased interest in converting waste by-products into valuable chemicals by using renewable electricity. Glycerol, co-generated from biodiesel production, is an abundant low-value feedstock, the valorisation of which could improve both the economics and sustainability of biofuel industries. Electrochemical glycerol oxidation is a promising route because it can operate under mild conditions while producing speciality chemicals used in cosmetics, pharmaceuticals, and food industry. However, industrial implementation remains limited because catalyst performance observed in laboratory studies often declines under practical operating conditions, where surface poisoning and the rate of mass transport strongly affect electrocatalytic performances. This implies that catalyst engineering alone is insufficient for scale-up, and that reactor engineering and operational strategies are equally important. Our findings show that glycerol electrooxidation on platinum is sluggish due to catalyst poisoning rather than by glycerol transport limitations. We developed and optimised pulsed potential strategy which can continuously regenerate platinum active sites, sustaining current densities for long-term operation. When combined with rotatory operation, forced convection further improved performance and influenced product selectivity. This work demonstrates that coupling dynamic catalyst regeneration with convective transport control can significantly improve catalyst stability, activity, and selectivity without complex material redesign.

## Introduction

To reduce reliance on fossil fuels, biodiesel production from renewable feedstocks such as vegetable oil and animal fats have expanded worldwide, generating glycerol as a major byproduct of the transesterification process.^1^ However, biodiesel is currently not commercially competitive with fossil fuel-derived diesel, largely due to limitations in feedstock availability. One potential strategy for improving the economic viability of biodiesel production is to valorise the low-value glycerol into higher-value chemicals. Among the various catalytic routes, the electrocatalytic conversion of glycerol represents a clean and sustainable option, as it can be coupled with cathodic reactions such as CO reduction,^2^ CO_2_ reduction,^3^ or the hydrogen evolution reaction,^4^ while being driven by renewable electricity. By adjusting operational conditions such as the electrocatalyst composition, electrolyte pH, applied potential, and temperature, the glycerol electrooxidation reaction (GOR) can be tuned to selectively produce high-value products such as glyceric acid, glyceraldehyde, lactic acid and formic acid.^5^ Among these, lactic acid is considered the most economically viable and is widely used in the production of biodegradable thermoplastics, with the market projected to reach $6654 million by 2033.^6,7^

Progress towards commercialization has been facilitated by employing zero-gap membrane electrode assembly (MEA)-based flow electrolysers for GOR, to achieve industrially relevant current densities (200–1200 mA cm^−2^).^8^ In these systems, an ion-exchange membrane is sandwiched between two catalyst-coated electrodes to form the MEA. Electrodes are typically supported on porous gas diffusion layers (GDLs), which serve as an interface between the catalyst layers and the flow field plates. The GDLs facilitate efficient reactant transport, removal of products, water management, and electronic conductivity. However, such a configuration is not well suited for studying intrinsic reaction kinetics and mechanisms due to the complex coupling between reaction kinetics, ohmic losses, and mass transport limitations. The presence of heterogeneous mass transport and non-uniform potential distributions across multiple layers of MEA makes the interpretation of the kinetics of the various (electro) chemical reactions particularly challenging.^9^ To obtain decoupled information and accurately probe the intrinsic reaction kinetics of newly developed catalysts, well-defined mass transport systems incorporating rotating electrodes such as the rotating disk electrode (RDE)^10^ and rotating cylinder electrode (RCE)^11^ are employed. The RDE enables precise control of mass transport through forced convection generated by electrode rotation, facilitating the decoupling of kinetic and mass-transport contributions and thereby allowing accurate evaluation of the activity and stability of novel catalysts. Typically, milligram-scale quantities of catalyst are deposited onto the planar surface of a conductive disk (*e.g.*, platinum, gold, or glassy carbon) embedded within an insulating holder.^12^ However, translating catalyst performance from RDE measurements to MEA configurations has remained a critical challenge. Catalysts that demonstrate high activity in RDE measurements frequently underperform in MEA electrolysers, highlighting the limited reliability of RDE-derived metrics for industrially relevant conditions.^9^ RDE systems also complicate kinetic studies of multi-electron transfer processes due to the concentration gradient that emerges along the radial direction of the disk.^13^ This discrepancy motivates the development of improved device architectures and evaluation approaches that maximize electrocatalytic performance while minimizing system complexity.

Despite extensive efforts to develop state-of-the-art catalysts with complex architectures capable of delivering industrially relevant current densities in realistic continuous-flow MEA electrolysers or fuel cells,^14,15^ the activity and product distribution of GOR remains highly sensitive to experimental conditions.^16^ GOR involves a highly complex reaction network arising from multiple adsorption configurations, molecular interactions at active sites, and the coexistence of faradaic and non-faradaic pathways, resulting in a broad distribution of products and transient intermediates.^17^ In particular, it depends strongly on electrode potential, temperature, electrolyte composition,^18^ and mass-transport conditions,^19,20^ as well as on electrode and catalyst characteristics, including catalyst loading^21^ and surface roughness.^22^ Disentangling the respective contributions of species-specific mass transport and intrinsic surface kinetics is critical, as GOR generates soluble intermediates that may desorb from the catalyst surface, diffuse into the electrolyte, or re-adsorb for subsequent oxidation.^23,24^ Consequently, elucidating the mechanistic role of mesoscopic mass transport is essential for understanding and controlling reaction pathways. For example, Melle *et al.* demonstrated that the rotation rate of a platinum RDE affects the removal of glyceraldehyde (GLAD), an early intermediate in GOR, which was identified as a primary contributor to platinum (Pt) surface poisoning in acidic media.^20^

Among the various electrocatalysts studied, Pt-based materials display the highest activity for glycerol electrooxidation, offering high current densities at relatively low overpotentials over a broad pH range compared with other noble metals.^5,17^ Nevertheless, their performance is often limited by rapid surface poisoning from strongly adsorbed CO (*CO) and other glycerol-derived intermediates, which block active sites and lead to catalyst deactivation, particularly in alkaline media, where competition with hydroxide ion (OH^−^) adsorption is more pronounced according to eqn (1).^25^ The adsorption and initial dehydrogenation of glycerol on Pt(111) have been analyzed in detail by Meng *et al.*^26^ Glycerol preferentially adsorbs *via* carbon atoms (*C), involving either the central carbon, the terminal carbons, or both. Subsequent dehydrogenation of the adsorbed glycerol species (*C_3_H_8_O_3_) proceeds according to eqn (2), while incorporation of surface adsorbed-hydroxyl (*OH) according to eqn (3) generates more oxidized surface intermediates:

1 OH^−^ + * → *OH + e^−^

2 *C_3_H_8_O_3_ → *C_3_H_7_O_3_ + H^+^_(aq)_ + e^−^

3 *C_3_H_8_O_3_ + 2*OH → *C_3_H_6_O_3_ + 2H_2_O_(1)_ + 2* + 2e^−^

To mitigate poisoning and improve catalyst durability and selectivity, considerable effort has been devoted to rational catalyst design and the optimization of electrochemical operating conditions. Strategies for enhancing the performance of noble metal-based electrocatalysts include tailoring nanostructure and morphology,^27^ alloying Pt or Au with other metals,^28–31^ Pt surfaces with p-block adatoms,^32–35^ and employing functional supports such as metal oxides with acidic surface sites.^36–38^ Since the reduction of Pt oxides and removal of strongly bound species such as *CO and glycerol derived-intermediates can restore active surface sites and sustain glycerol oxidation, the implementation of periodic alternating potential pulse strategies have also attracted attention.^39^ In particular, intermittent chronoamperometry or pulse potential methods have been used to modulate the accumulation and removal of poisoning species.^40–42^ Chen *et al.* developed a high and low potential pulsing strategy that can mitigate the deactivation of the Pt catalyst due to the accumulation of Pt oxides and glycerol-derived intermediates on the catalyst surface such as glyceraldehyde (GLAD) and glyceric acid (GLA).^41^ Under optimal pulsed electrolysis conditions, high oxidation rate of GLAD due to high coverage of *OH at high potential pulse (0.7 V_RHE_, for 0.05 s) and fast desorption of GLA and removal of Pt oxides at low potential pulse (0.3 V_RHE_, for 0.5 s) facilitated the reactivation of active sites for the re-adsorptions of glycerol and OH^−^, resulting in the improvement of stability and higher selective production of glyceric acid (81.8%) than constant potential electrolysis at 0.7 V_RHE_ (37.8%). Roquet *et al.* employed a pulsed potential protocol comprising three sequential steps: a glycerol oxidation step at 0.79 V_RHE_ for 10 s, followed by two short pulses at 1.8 and 0.45 V_RHE_ applied for 0.1 and 0.2 s, respectively.^42^ The first pulse at 1.8 V_RHE_ served to oxidize surface-bound poisoning intermediates generated during the application of glycerol oxidation potential, while the subsequent pulse was applied to reduce the surface oxide layer formed during the preceding high-potential step.

However, to date, few studies have addressed the simultaneous maintenance of high activity and stability while explicitly accounting for the influence of forced convection during glycerol electrooxidation, particularly those employing planar Pt electrodes as model catalysts under well-defined hydrodynamic conditions. Herein, we developed a custom divided three-electrode electrochemical cell (Scheme 1), incorporating a rotating cylindrical polycrystalline Pt electrode (Pt RCE), based on a design originally introduced by Morales-Guio and co-workers.^43,44^ This configuration enables systematic investigation of the impact of electrolyte convection on the electrocatalytic performance of Pt during GOR. Moreover, the relatively high ratio of geometric electrode surface area to electrolyte volume (0.033 cm^−1^) increases sensitivity for liquid product analysis, allowing the generation of detectable amounts of glycerol oxidation products in hour-long electrolysis. We also present a systematic study to develop the optimal potential pulsing strategy to dynamically regulate the interface between the Pt RCE and the glycerol-containing electrolyte to improve the electrocatalytic performance for GOR.

**Scheme 1:**
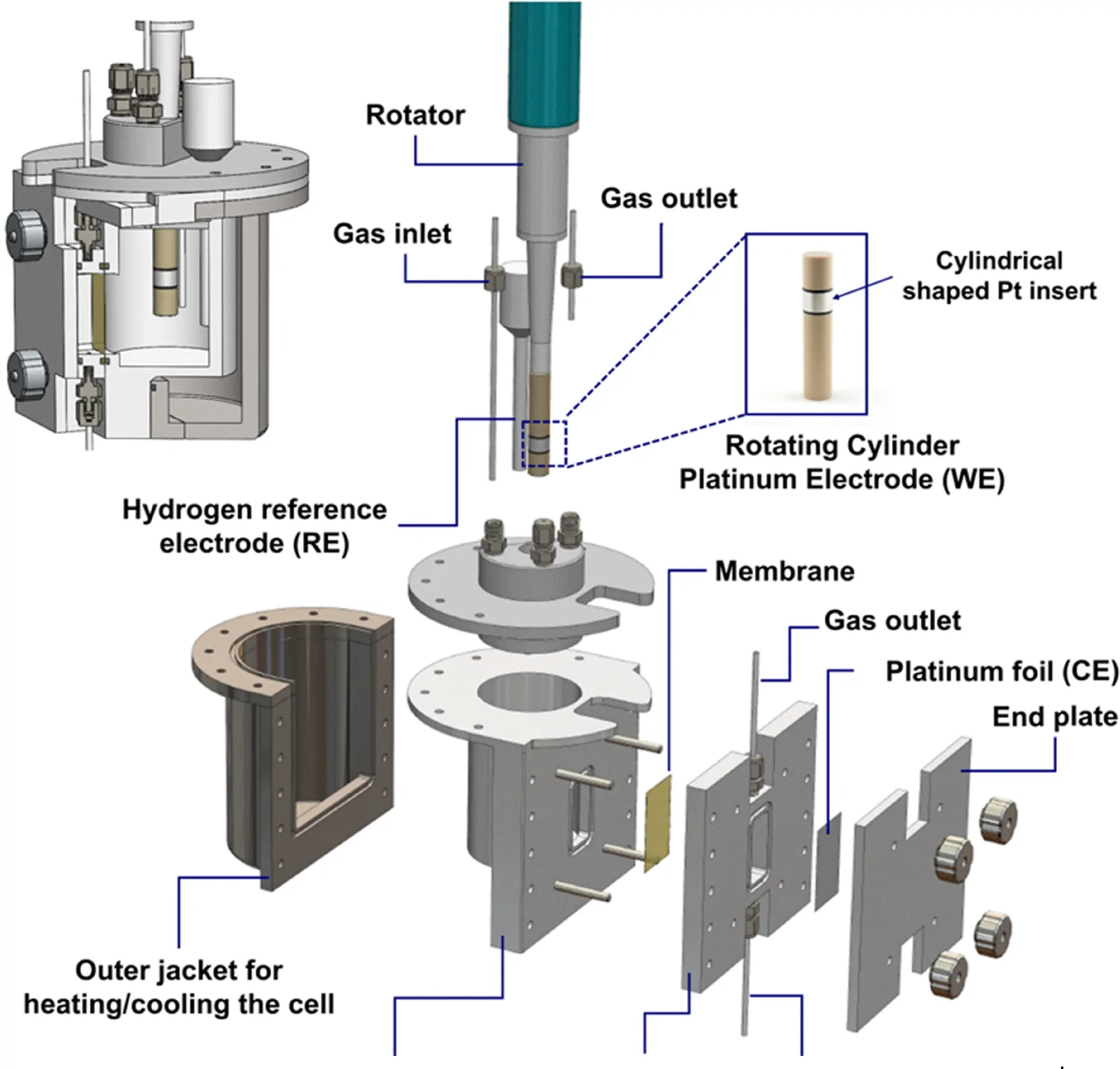
Schematic of the rotating cylinder electrode cell design.^43,44^

## Results and discussion

### Electrochemical characterization

The preparation of the Pt RCE through polishing, cleaning and flame annealing is described in the Experimental details in the SI. The cleanliness of the Pt RCE surface and cell were initially verified by cyclic voltammetry (CV) in Ar-saturated 0.1 M NaOH solution (pH ∼ 13) at room temperature (∼20 °C), scanning the applied potential between 0.05 and 1.1 V_RHE_ at 50 mV s^−1^ with a rotation rate of 1000 rpm (see inset, Fig. 1b). The cell temperature was controlled by circulating hot water through the outer jacket, raising the electrolyte temperature in the working electrode compartment. After the electrolyte temperature was increased to 60 °C, 180 µL of the 10 M glycerol stock solution was added to the 0.1 M NaOH solution to achieve a final concentration of 0.02 M resulting in a decrease of the open-circuit potential (OCP) from approximately 0.8 to 0.3 V_RHE_ (Fig. 1a). After the system reached equilibrium and no electrode rotation was applied, the value of OCP was 0.3 ± 0.03 V_RHE_, indicating the possible minimum potential at which glycerol spontaneously reacts with the Pt electrode surface. Therefore, the potential of 0.3 V_RHE_ was used as an auxiliary potential to promote the re-adsorption of glycerol in the potential pulsing protocol.

**Fig. 1:**
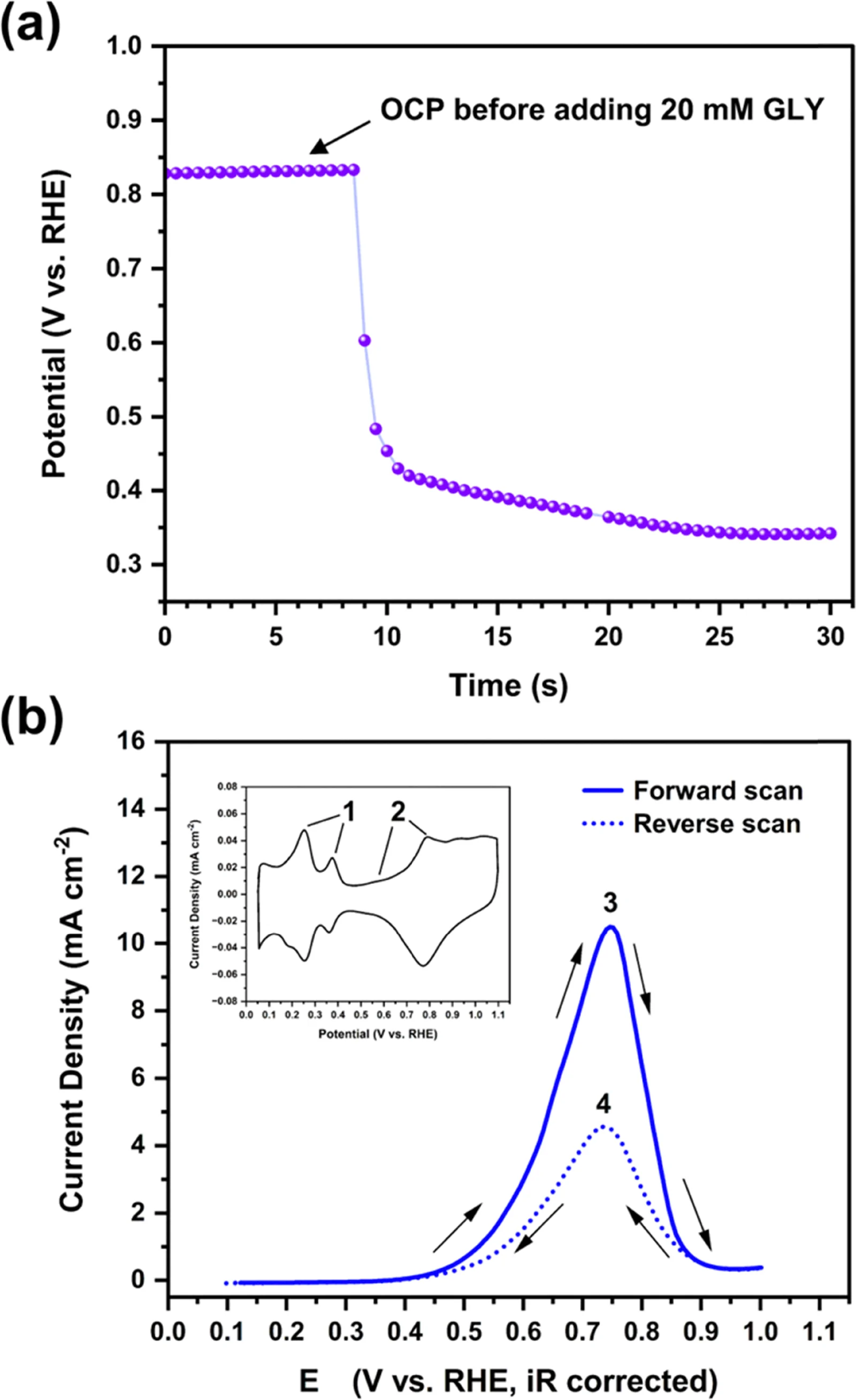
(a) Open-circuit potential measurement during the addition of glycerol in 0.1 M NaOH. (b) The 10th cycle of CV recorded at a scan rate of 50 mV s^−1^ and an electrode rotation rate of 1000 rpm in 0.1 M NaOH and 0.02 M glycerol at 60 °C (inset: in blank 0.1 M NaOH at room temperature).

Subsequently, ten consecutive cyclic voltammetry (CV) scans were recorded under quiescent conditions at a scan rate of 50 mV s^−1^ with online iR-drop compensation (Fig. 1b). During the first positive-going scan in the presence of glycerol, the electrode potential was held at 0.05 V_RHE_ for 10 s before being swept from 0.05 to 1.0 V_RHE_. The characteristic oxidative features between 0.1 and 0.4 V_RHE_, associated with *H desorption on Pt^45^ (see inset, Fig. 1b, peak 1), were no longer observed, indicating that adsorbed glycerol occupied the surface-active sites and suppressed hydrogen adsorption.^46,47^ Upon further positive polarization, the onset of glycerol oxidation appeared at approximately 0.5 V_RHE_, with the oxidation current reaching a maximum at ∼0.75 V_RHE_ (Fig. 1b, peak 3). In the potential region between 0.5 and 0.75 V_RHE_, the rising current was attributed to the formation of surface *OH species from water activation (see inset, Fig. 1b, peak 2), which promoted the oxidation of carbonaceous intermediates through a bifunctional mechanism.^48,49^ When the potential was swept beyond 0.75 V_RHE_, a rapid decrease in the oxidation current was observed up to 1.0 V_RHE_. This decline cannot be ascribed to mass-transport limitations, which were probed over a range of RCE rotation rates (see Fig. S5 and Table S3 for more detail about hydrodynamic analysis of GOR on RCE), but instead results from two coupled surface-deactivation processes: (i) the accumulation of strongly adsorbed poisoning species, such as *CO or formyl-like intermediates, and (ii) the formation of strongly bound Pt–OH and/or PtO_*x*_ species that block metallic Pt^0^ sites required for glycerol adsorption.^50^ The combined effects of surface oxidation and intermediate poisoning, therefore, inhibit further glycerol oxidation at high potentials. This interpretation is supported by the reappearance of a glycerol oxidation peak at a similar potential during the reverse (negative going) scan from 1.0 V_RHE_ (Fig. 1b, peak 4).

The reverse-scan peak is attributed to the oxidation of residual glycerol or adsorbed intermediates, enabled by the partial regeneration of active sites following the reductive desorption of *OH.^35,51^ Moreover, the difference in peak current densities between the forward and reverse scans reflects the extent of intermediate accumulation and surface site recovery.^52^ Upon sweeping the potential back to 0.05 V_RHE_ and repeating the scan, glycerol oxidation occurred within the same potential window, although with a reduced peak current density (see Fig. S5d for a comparison of the peak current density between the 1st and 10th CV cycles at 1000 rpm). This gradual decrease could be attributed to the incomplete removal of strongly adsorbed species accumulated during previous cycles.^53^ Beyond activity assessment, these CV results provide important mechanistic insights into the dynamic deactivation and regeneration of the Pt catalyst surface during glycerol electrooxidation.

### Pulsed-potential electrolysis of glycerol

The pulsed electrolysis in this work was established by adapting the catalyst surface regeneration concept obtained from CV results. The three potentials, including 0.3, 0.75, and 1.0 V_RHE_, were chosen and used in the sequence analogous to the sweeping potentials in CV. The pulsing protocol was initiated with the oxidation of glycerol at an applied potential of 0.75 V_RHE_. The subsequent sequential application of two auxiliary potentials (1.0 V_RHE_ and 0.3 V_RHE_) mitigated the deactivation of the Pt catalyst, which otherwise caused an exponential decay in the glycerol oxidation current. The high potential of 1.0 V_RHE_ resulted in the displacement of the carbonaceous poisoning species by *OH. The low potential of 0.3 V_RHE_ regenerated the surface to enable glycerol re-adsorption. The purpose of each potential step is shown schematically in Scheme 2. The duration of each step was explored as a variable (Fig. S6–S9 and Table S1). The application of either 0.3 V_RHE_ (Fig. S10) or 1.0 V_RHE_ (Fig. S11) as the only auxiliary potential was also investigated to understand the regeneration process of the catalyst surface.

**Scheme 2:**
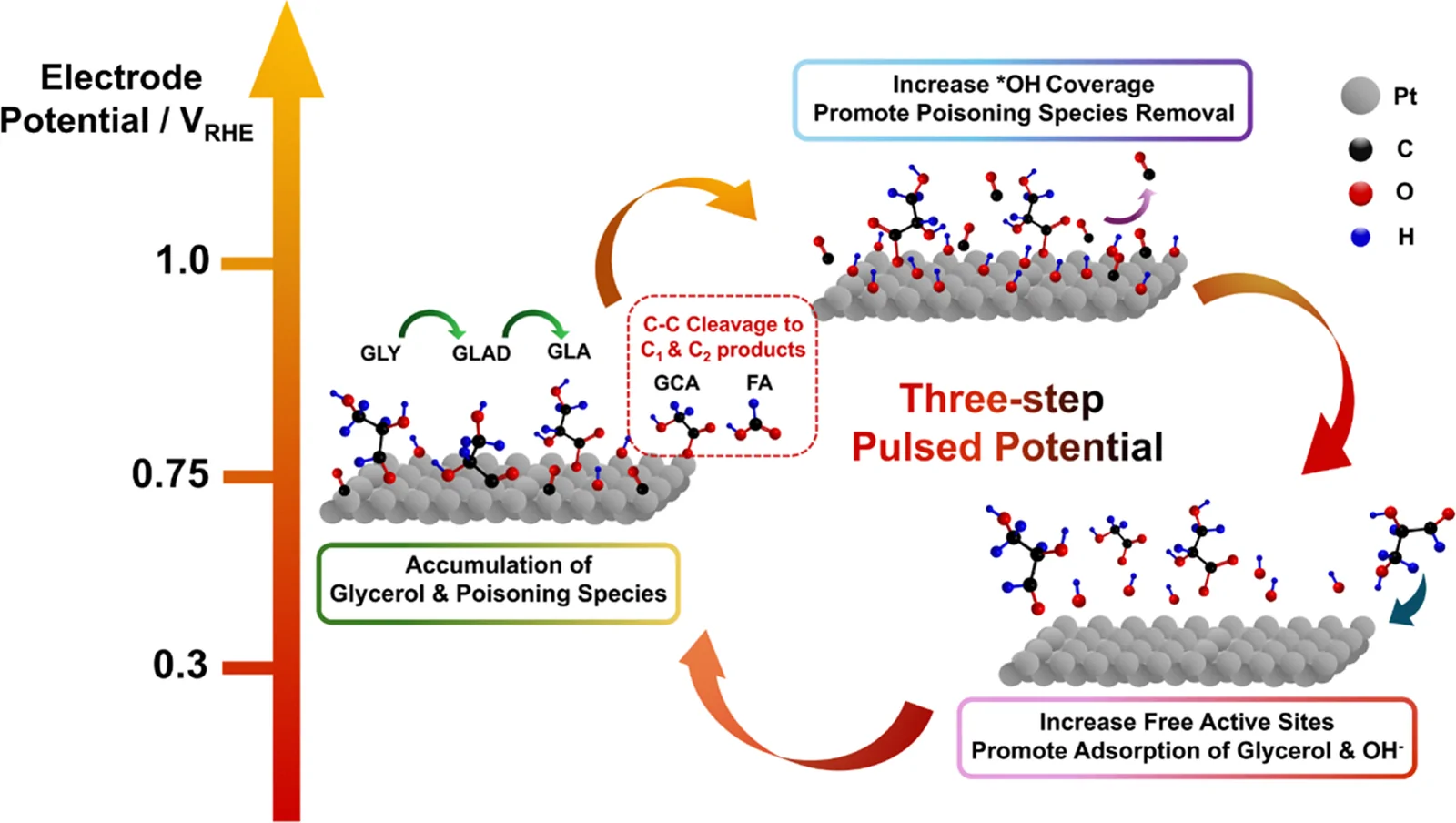
Schematic illustration of the three-step pulsed potential process for GOR on Pt catalyst in alkaline media.

The effect of pulse duration was systematically examined and optimized for both the three-step and two-step pulsed potential protocol. For comparison, the current responses from the first five cycles of each pulsing protocol were evaluated using identical pulse duration (*t*_0.75V_ = *t*_1.0V_ = *t*_0.3V_ = 1 s), as shown in Fig. 2a–c. The three-step pulsed potential protocol exhibited a pronounced enhancement in catalyst surface regeneration (Fig. 2d). In addition, the current density and total charge passed over 20 minutes were compared for different three-step pulsing conditions by varying the duration of each potential step (*t*_0.75V_, *t*_1.0V_, *t*_0.3V_), with the results summarized in Table S1 and Fig. S7–S9. Notably, shorter glycerol oxidation step durations (*t*_0.75V_ = 0.5 s; P1–P9; Fig. S7) yielded higher average current densities but lower total charge, attributable to the reduced duty cycle (*d*) at 0.75 V_RHE_ (see Experimental methods for duty cycle calculation). Moreover, extending *t*_0.3V_ relative to *t*_0.75V_ led to a modest increase in the average current density, indicating that allocating at least twice the resting duration at 0.3 V_RHE_ is beneficial for maximizing catalyst regeneration (Fig. S7–S9(a–c)). This strategy was further corroborated by the two-step pulsed potential experiments, where longer *t*_0.3V_ combined with shorter *t*_0.75V_ hindered the accumulation of poisoning species (Fig. S10a and f). Conversely, extending *t*_1.0V_ of the two-step pulsed potential protocol (0.75 and 1.0 V_RHE_) did not promote glycerol re-adsorption, as a greater fraction of active sites remained blocked by oxygenated species and were not regenerated (Fig. S11a–c). In contrast, within the three-step pulsed potential protocol, longer *t*_1.0V_ increased the average current density, particularly when a prolonged glycerol oxidation step was employed (Fig. S7–S9d–f).

**Fig. 2:**
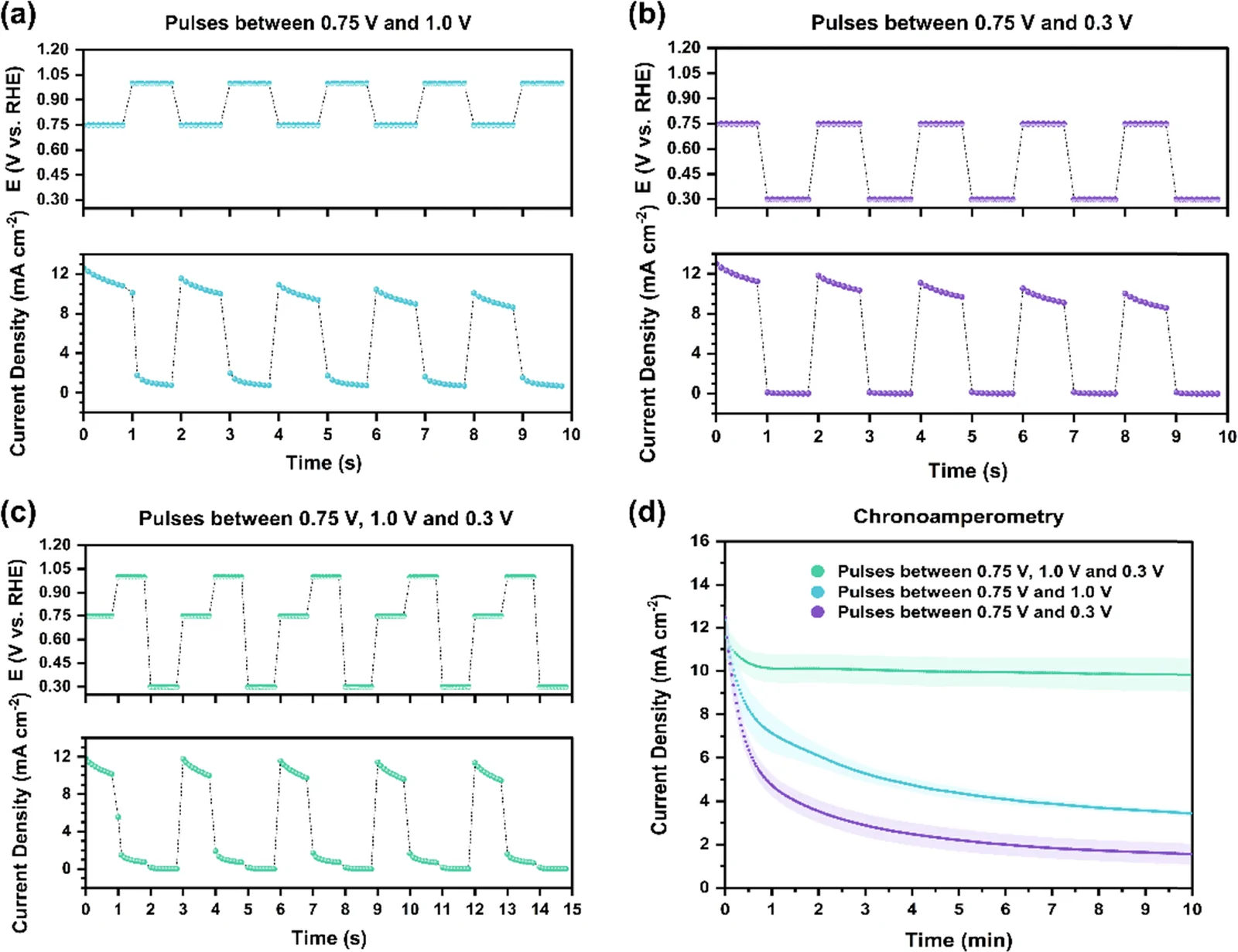
Waveform of pulsed potential and current responses in the first five cycles for the case of (a) 0.75 V_RHE_ and 1.0 V_RHE_, and (b) 0.75 V_RHE_ and 0.3 V_RHE_. (c) 0.75 V_RHE_, 1.0 V_RHE_, and 0.3 V_RHE_. (d) Comparison of the average current density during 0.75 V_RHE_ for the three-step and two-step pulses in 0.02 M glycerol and 0.1 M NaOH over 10 min at 60 °C under static conditions (no electrode rotation); Three independent experiments were performed, and the corresponding standard deviations are shown as a shaded color band.

To validate the effectiveness of the pulsed potential strategy, 2 hour glycerol electrolysis experiments were independently conducted under a constant potential (0.75 V_RHE_), a two-step pulsed potential protocol (*t*_0.75V_ = 1 s, *t*_1.0V_ = 1 s), and a three-step pulsed potential protocol (P14: *t*_0.75V_ = 1 s, *t*_1.0V_ = 1 s, *t*_0.3V_ = 1 s). The three-step pulsed potential protocol exhibited superior glycerol oxidation rates and stability compared to the constant-potential and two-step pulsed potential electrolysis, achieving an average current density during 0.75 V_RHE_ of 9 mA cm^−2^ and glycerol conversions that were 17- and 4-fold higher, respectively (Fig. 3a–c). The product distributions from glycerol oxidation under constant-potential and pulsed-potential conditions were analyzed by high-performance liquid chromatography (HPLC). Owing to the relatively low glycerol conversion achieved under the constant-potential and two-step pulsed potential protocols, only small quantities of products were detected in the corresponding HPLC chromatograms (see Fig. S1 for chromatogram comparison). Fig. 3c shows that the implementation of the three-step pulsed potential protocol led to a pronounced shift in product selectivity: the faradaic efficiency toward lactate decreased from 32% under constant-potential operation to 3%, while glycerate became the dominant product with the faradaic efficiency of 37%. This result demonstrates that the faradaic oxidation of glyceraldehyde to glycerate was favored over non-faradaic pathways, thereby suppressing competing chemical transformations of glyceraldehyde, including its conversion to lactate.^54^

**Fig. 3:**
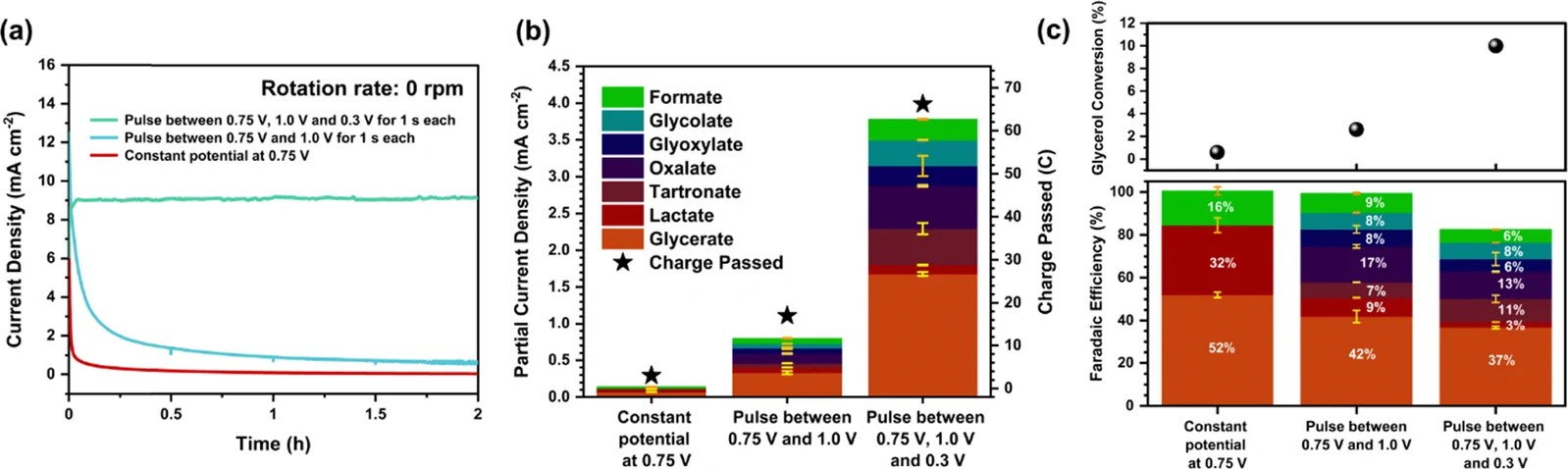
Glycerol electrooxidation performance after 2 hour electrolysis using constant potential of 0.75 V_RHE_, sa two-step pulsed potential protocol (0.75 V_RHE_ for 1 s and 1.0 V_RHE_ for 1 s), and a three-step pulsed potential protocol (P14: 0.75 V_RHE_ for 1 s, 1.0 V_RHE_ for 1 s, 0.3 V_RHE_ for 1 s) in 0.02 M glycerol and 0.1 M NaOH at 60 °C under static conditions (0 rpm): (a) average current density during 0.75 V_RHE_, (b) partial current density and charge passed and (c) faradaic efficiencies and glycerol conversion.

To further optimize the three-step pulsed potential protocol, 6 hour electrolysis experiments were conducted using different duty cycles. As the duty cycle at 0.75 V_RHE_ increased (*i.e.*, longer *t*_0.75V_), the average current density decreased from *ca.* 9 mA cm^−2^ at *d* = 33% to *ca.* 4 mA cm^−2^ at *d* = 80% (Fig. 4a). The charge passed and glycerol conversion increased with increasing duty cycle but declined when the duty cycle exceeded 50% (Fig. 4b and c). This decrease is attributed to the progressive accumulation of poisoning intermediates, which suppresses the glycerol oxidation rate at higher duty cycles. Accordingly, three-step pulsed potential protocols with duty cycles below 50% were examined in greater detail (Fig. S15–S19). Both protocols operated at a 25% duty cycle (P2 and P15) exhibited a modest increase in average current density to *ca.* 10 mA cm^−2^ (Fig. S15) albeit with slightly lower glycerol conversion (*ca.* 17%) compared to the 33% duty cycle protocol (P1, *ca.* 20%; Fig. S16). Overall, the corresponding product selectivity did not vary significantly across these pulsing protocols (Fig. S14 and S19); however, when considering both the faradaic efficiency toward glycerate and the effectiveness of catalyst surface regeneration, the 25% duty cycle emerged as optimal. In particular, protocol P2 (*t*_0.75V_ = 0.5 s; *t*_0.1V_ = 0.5 s; *t*_0.3V_ = 1 s) achieved a glycerate faradaic efficiency of 40%, representing the most favorable balance between activity, selectivity, and stability.

**Fig. 4:**
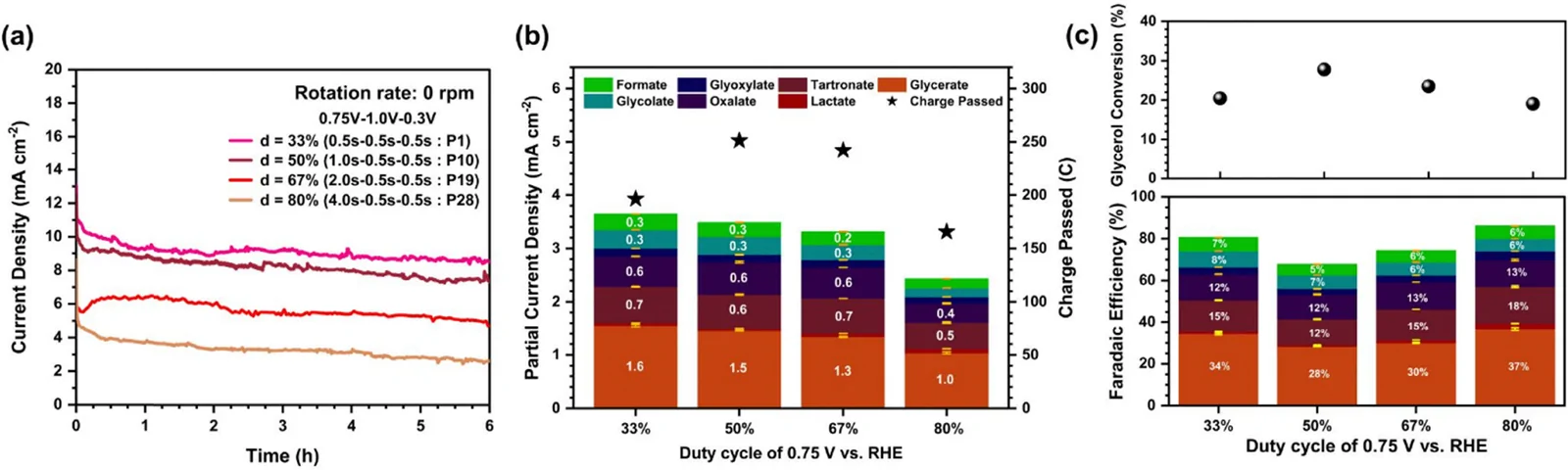
Glycerol electrooxidation performance after 6 hour electrolysis using three-step pulsed potential protocols with varying duty cycles. The duration of the glycerol oxidation step (*t*_0.75V_) was set to 0.5, 1.0, 2.0, and 4.0 s, while *t*_1.0V_ and *t*_0.3V_ were both fixed at 0.5 s, in 0.02 M glycerol and 0.1 M NaOH at 60 °C under static conditions (0 rpm): (a) average current density at 0.75 V_RHE_, (b) partial current densities and total charge passed and (c) faradaic efficiencies and glycerol conversion.

### Pulsed-potential electrolysis coupled with mass transport control

While the three-step pulsed potential protocol on a stationary Pt electrode effectively suppresses poisoning and promotes surface reactivation, the GOR rate is ultimately constrained by the mass transport of reactants and intermediates at the electrode–electrolyte interface. To probe the effect of mass transport on GOR activity at the Pt RCE, chronoamperometry at 0.75 V_RHE_ and cyclic voltammetry at a scan rate of 50 mV s^−1^ were performed at varying electrode rotation rates. Under constant-potential conditions, Fig. 5a shows that increasing the rotation rate decreased GOR activity, likely due to enhanced removal of glycerol and/or adsorbed intermediates that are necessary for further electrooxidation, thereby limiting the overall reaction rate. Conversely, CVs recorded under electrode rotation exhibited enhanced GOR activity, with higher peak currents and charge transfer between 0.4–1.0 V_RHE_ (Fig. 5b, c and Fig. S5), reflecting improved mass transport. Notably, at rotation rates above 500 rpm, forward–reverse peak differences increased due to stronger convection, which removes intermediates away from the electrode surface, thereby reducing the availability of oxidizable species on the cleaner surface during the reverse potential sweep (Fig. S5c–f). In contrast, a mild rotation rate of 100 rpm provided the most favourable enhancement by mass transport, as the reverse scan consistently matched the forward scan in peak current density and charge transfer. This behavior suggests that mild convection optimally balances glycerol replenishment and catalyst surface reactivation, enabling more efficient GOR during the reverse sweep. Intermediate convections improve mass transport to and from the electrode while allowing for enough residence time of intermediates in the proximity of the electrode.^55^ These findings indicate that the interplay between convective electrolyte transport and surface reactivation defines a multi-variable space for tuning both catalytic activity and product selectivity.

**Fig. 5:**
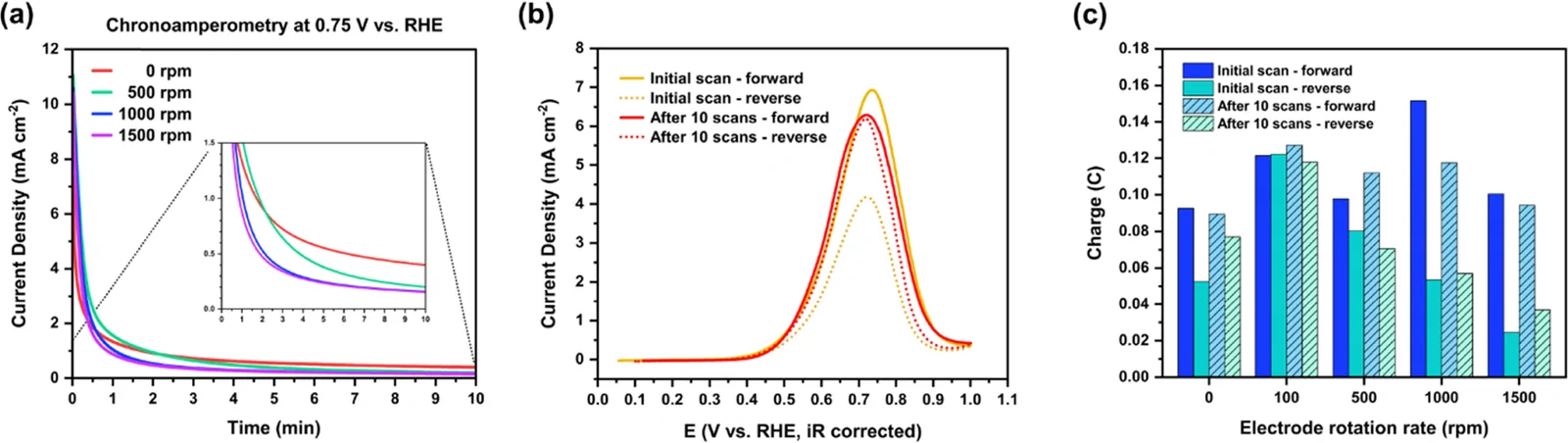
Glycerol electrooxidation performance in 0.02 M glycerol and 0.1 M NaOH at 60 °C as a function of electrode rotation rate. (a) Chronoamperometric response at 0.75 V_RHE_ (inset: enlarged view of the current density over 10 minutes). (b) Cyclic voltammogram recorded at 50 mV s^−1^ and 0 rpm. (c) Charge transfer within the 0.4–1.0 V_RHE_ potential window extracted from the CVs.

The three-step pulsed potential protocol operated at a 25% duty cycle, P15 (*t*_0.75V_ = 1 s; *t*_1.0V_ = 1 s; *t*_0.3V_ = 2 s), was also used to investigate the influence of electrode rotation rate. As shown in Fig. 6a, the three-step pulsed potential electrolysis over 6 hours exhibited mass-transport dependence that is consistent with the trends observed in the CV results, with the exception of those recorded at 0 rpm. All electrode rotation rates enhanced the average current densities relative to the stationary case at 0 rpm, with the most pronounced improvement achieved under mild convection at 100 rpm, yielding the highest average current density of *ca.* 14 mA cm^−2^. However, excessive convection at rotation rates above 500 rpm led to reduced improvements in both average current density and charge passed, indicating less favourable mass-transport conditions (Fig. 6a and b). As shown in Fig. 6c, enhanced activity was confirmed by a glycerol conversion rate nearly double (33–37%) that of the stationary electrode (17%). More importantly, electrode rotation markedly increased the yields of C_3_ products. In particular, lactate formation was strongly enhanced with increasing rotation rate, as reflected by an approximately tenfold increase in faradaic efficiency from 2% at 0 rpm to 20% at 1500 rpm (Fig. 6c and d). This enhancement can be attributed to the convective removal of soluble intermediates, particularly glyceraldehyde, from the catalyst surface at elevated rotation rates. Upon transport into the strongly alkaline electrolyte, glyceraldehyde preferentially undergoes non-faradaic base-catalyzed dehydration followed by a Cannizzaro rearrangement, leading to lactate formation, as illustrated in Fig. 6e.^56–58^ To confirm this proposed mechanism, the chemical stability test for glyceraldehyde under similar alkaline conditions at 60 °C was implemented in the absence of applied potential and glycerol. The HPLC chromatograms of the samples collected during 1 hour test (Fig. S25) show that the glyceraldehyde was sufficiently stable under such conditions to undergo chemical homogeneous conversions.

**Fig. 6:**
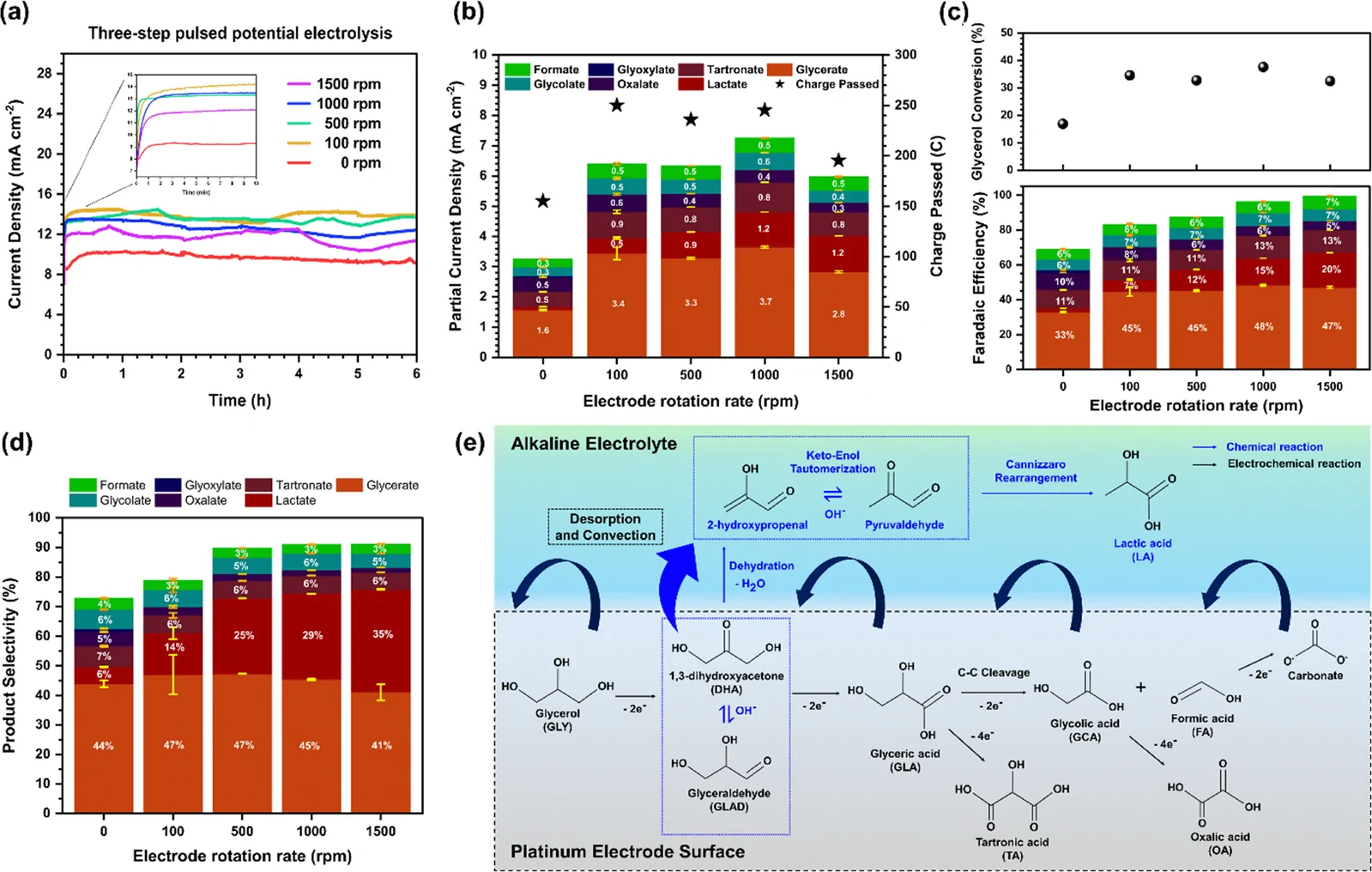
Glycerol electrooxidation performance in 0.02 M glycerol and 0.1 M NaOH at 60 °C as a function of electrode rotation rate: (a) Average current density over 6 hours obtained using the three-step pulsed potential protocol P15 (*t*_0.75V_ = 1 s, *t*_1.0V_ = 1 s, *t*_0.75V_ = 2 s); inset: enlarged view of the first 10 minutes, (b) partial current densities and charge passed, (c) faradaic efficiencies and glycerol conversion, and (d) product selectivity. (e) Schematic illustration of the proposed GOR mechanism on a rotating Pt electrode.

## Conclusion

This work establishes a well-defined mass transport system based on a rotating cylinder electrode (RCE), wherein the turbulent flow regime is more representative of industrially relevant conditions. The effect of electrode rotation rate on the glycerol electrooxidation performance of planar polycrystalline platinum was systematically investigated. The results confirm that glycerol electrooxidation is not limited by reactant mass transport to the electrode surface, but rather by sluggish surface processes, including the inherently slow kinetics of the multistep reaction, the formation of poisoning intermediates, and the limited adsorption rate of hydroxyl species. To overcome these limitations, a three-step pulsed potential protocol was developed, providing an effective and stable strategy to mitigate Pt surface poisoning during glycerol electrooxidation. This approach facilitates the oxidative removal of strongly adsorbed intermediates at 1.0 V_RHE_, followed by the regeneration of active sites through reduction of surface oxides at 0.3 V_RHE_. Notably, longer resting periods at 0.3 V_RHE_ relative to the glycerol oxidation step at 0.75 V_RHE_ were found to maximize catalyst regeneration, delivering average current densities up to *ca.* 12 mA cm^−2^. Crucially, this pulsed strategy alters the rate-limiting step of glycerol electrooxidation, whereas constant-potential operation is dominated by surface poisoning and deteriorates under enhanced mass transport. Pulsed electrolysis benefits from forced convection, enabling improved catalytic performance under turbulent flow conditions. By integrating optimized pulsing with controlled hydrodynamics using a rotating Pt cylinder electrode, lactate selectivity increased from 6% at 0 rpm to 35% at 1500 rpm at 12 mA cm^−2^. Beyond interfacial phenomena at the electrode–electrolyte interface, these results highlight the critical role of bulk-phase processes in governing glycerol conversion and product selectivity. The outcomes of this work have important implications for scaling up glycerol electrolysis to practical industrial levels, where variations in reactor geometry and operating conditions – and consequently mass transport behaviour – can significantly influence product distribution. To further enhance the industrial relevance of this approach, future efforts should focus on replacing Pt with non-noble metal RCE systems, such as Ni- or Co-based electrocatalysts, offering a pathway toward more economically viable and poisoning-resistant electrolyser technologies.

## Experimental methods

### Chemicals

Glycerol (ACS reagent, ≥99.5%, Sigma-Aldrich), sodium hydroxide solution (30%, Suprapur®) and sulfuric acid (≥98%, EMSURE® for analysis, Supelco®) were used to prepare electrolyte solutions. The following chemicals were used as reference glycerol oxidation products for HPLC calibration: formic acid (Reagent grade, ≥95%, Sigma-Aldrich), glycolic acid (for synthesis, ≥99%, Sigma-Aldrich), glyoxylic acid monohydrate (98%, Sigma-Aldrich), oxalic acid dihydrate (ACS reagent grade, ≥99%, Sigma-Aldrich), tartronic acid (97%, Sigma-Aldrich), lactic acid (85–90% Sigma-Aldrich) and dl-glyceric acid (20% in water, Sigma-Aldrich). All solutions were prepared using Milli-Q-water with a resistivity of 18.2 MΩ cm (Millipore Corp.) at room temperature.

### Electrochemical cell

All electrochemical measurements were performed in a custom membrane-separated cell made in-house using polyvinylidene fluoride (PVDF) for the body and polytetrafluoroethylene (PTFE) for the cap. This three-electrode electrochemical cell resembles the modified design introduced by Morales-Guio and co-workers,^43,44^ which used a compression-type connection between the counter electrode compartment and working electrode compartment to reduce the overall cell resistance by shortening the distance between the working and counter electrodes and by enlarging the area of the membrane separating both compartments, as shown in the exploded view of the cell design (Scheme 1). To prevent a crossover of oxidized glycerol products from the working electrode chamber to the counter electrode chamber, a Nafion™ 115 membrane (The Chemours Company) was used as the cation exchange membrane; it was placed between the flanges on the working electrode compartment and on the counter electrode plate. Subsequently, a platinum foil (0.025 mm thick, 50 × 50 mm, 99.9% metal basis, Fisher Scientific) counter electrode was placed to cover the flange on another side of the counter electrode plate. One end of a copper tape (0.035 mm thick, 10 mm wide, RS PRO) was attached to the platinum foil, and another end protruded from the gap between the end plate and the counter electrode plate for an electrical connection. The chambers on the working electrode side and the counter electrode side can hold maximum electrolyte volumes of 150 mL and 10 mL, respectively.

The cell was equipped with the assembled Pt RCE and reversible hydrogen reference electrode (RHE) (HydroFlex®) by inserting the electrodes through the holes on the PTFE cap of the working electrode compartment. The position of the RCE tip was aligned with the center of the Pt foil counter electrode. The stem of the RHE reference electrode is 80 mm long, allowing its tip to be close to the Pt RCE to minimize the iR drop between the working and reference electrodes. The cap had three threaded male ports. One of them had a relatively larger diameter for adding glycerol, collecting electrolyte aliquots for analysis during measurement, or measuring the temperature of the electrolyte. The second port was used for inserting a PTFE pipe to purge the electrolyte with argon gas to remove dissolved O_2(aq.)_ from the electrolyte, and the excessive gases in the headspace could be removed through the last port.

Before inserting 8 mL of the prepared electrolyte solution into the counter electrode chamber, nitrogen gas was fed continuously through the bottom threaded port in the counter electrode plate and allowed to exit through the top port. The nitrogen gas flow rate was controlled at 20 SCCM (standard cubic centimeters per minute) by a mass flow controller (Bronkhorst®). Subsequently, 8 mL and 90 mL of the prepared electrolyte were added to the counter and working electrode chambers, respectively. In this work, the outer stainless-steel jacket enclosing the working electrode compartment was designed to enable electrolyte temperature control, as shown in Fig. S2. This modification is specifically for accurate temperature control of the glycerol oxidation reaction, the activity of which can be enhanced at elevated temperatures. Water from the heated water reservoir was pumped through the inlet at the bottom of the jacket, and subsequently, the overflow was pushed out from the top outlet to recirculate the heating. All experiments were carried out at 60 °C, and the temperature of the electrolyte inside the working electrode chamber was measured by using a thermometer at intervals.

### Electrochemical measurements

All electrochemical measurements were conducted using an Autolab PGSTAT302N potentiostat (Metrohm) equipped with a true linear scan generator (SCAN 250) module for accurate measurement of the fast transient behaviour of hydrogen adsorption/desorption, and with a FRA32 module for electrochemical impedance spectroscopy (EIS). The rotation speed of the RCE was controlled by the Autolab motor control unit.

To determine the ohmic potential drop (iR-drop resistance), potentiostatic electrochemical impedance spectroscopy (PEIS) was carried out at 0.3 V_RHE_ (open-circuit potential), over the frequency range from 10 000 Hz to 0.1 Hz with an amplitude of 10 mV_RMS_. Fig. S4 shows the Nyquist plot of the Pt RCE in 0.1 M NaOH + 0.02 M glycerol solution at 60 °C and the Randles circuit (equivalent electrical circuit) fitted by the electrochemical circle fit command provided in NOVA 2.1.8 software. Ohmic resistance (*R*_u_) of 2.6 Ω was obtained and used for iR compensation. To avoid oscillations, 85% of the *R*_s_ value was applied for the iR compensation function of the potentiostat during the measurement. Subsequently, 15% of the *R*_u_ was used to correct the measured potential (*V*_measured_) mathematically as follows: *V*_corrected_ = *V*_measured_ − (*i*_measured_ × 15% *R*_u_).^59^

All pulsed potential experiment data were recorded every 0.1 s. For the purpose of simplifying the comparison between different cases, only the averaged currents of the applied potential at 0.75 V_RHE_ are presented. The duty cycle (*d*) of glycerol oxidation at 0.75 V_RHE_ was calculated using the following formula:4
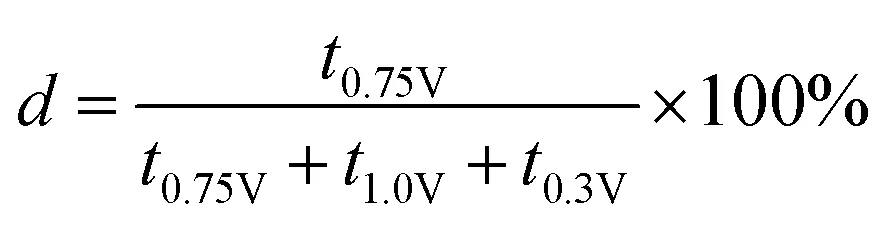
where *t*_0.75V_ is the duration of glycerol oxidation potential at 0.75 V_RHE_, *t*_1.0V_ is the duration of high oxidation potential at 1.0 V_RHE_, *t*_0.3V_ is the duration of resting potential at 0.3 V_RHE_. The protocols employed in this work are detailed in Table S1.

### Liquid oxidation product analysis

High-performance liquid chromatography (HPLC, Agilent 1260 Infinity II LC system) was used to quantify the concentration of liquid products produced by the electrolysis of glycerol. The system was equipped with a single separation column containing a stationary phase (Aminex HPX-87H column, Bio-Rad Laboratories, Inc) kept at a temperature of 60 °C. The mobile phase was 5 mM H_2_SO_4_ solution in 18.2 MΩ cm water. All HPLC measurements were conducted by using an isocratic pump with a flow rate of 0.5 mL min^−1^. The refractory index (RI) detector was used to quantify the concentration of glycerol at 35 °C. The ultraviolet (UV) detector at 210 nm was used to quantify the concentration of glycerol oxidation reaction products, including oxalic acid, tartronic acid, glyoxylic acid, glyceric acid, glycolic acid, lactic acid, and formic acid. Aliquots (400 µL) from each glycerol electrolysis experiment from both counter and working electrode chambers were collected after 2 and 6 hours of electrolysis and were diluted with 400 µL of 0.051 M H_2_SO_4_ to acidify the liquid samples to accommodate the pH requirement of the column (pH 1 to 3). Standard solutions of the expected glycerol oxidation products were prepared in a range of representative concentrations by dissolving the respective compounds in 0.05 M NaOH and 0.0255 M H_2_SO_4_ solution to construct the calibration curve. The integrated area under the HPLC peak of each compound was plotted against the concentration. All calibration curves used for product analysis are shown in Fig. S12.

The conversion of glycerol and selectivity for each product were calculated as follows:5
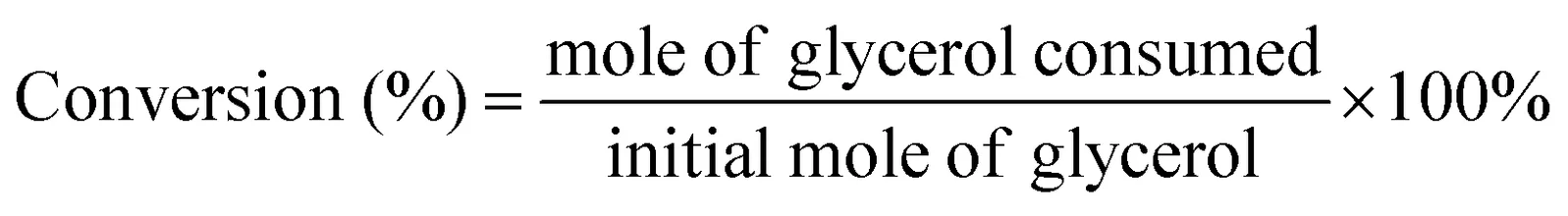
6
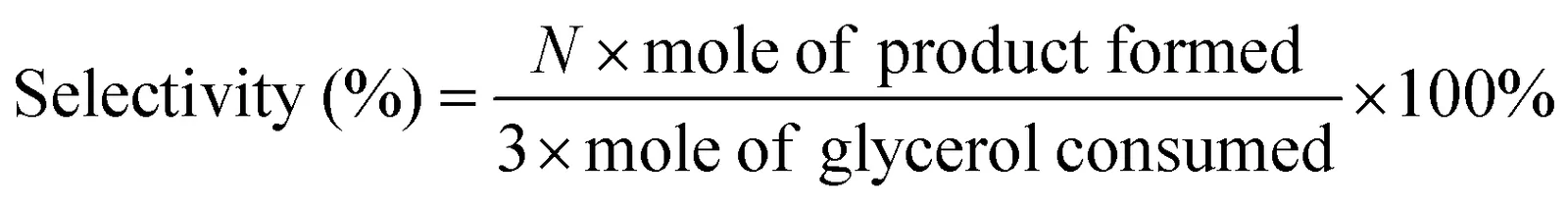
where *N* is the number of carbon atoms in the product.

Faradaic efficiency (FE) and partial current density were calculated as follows:7
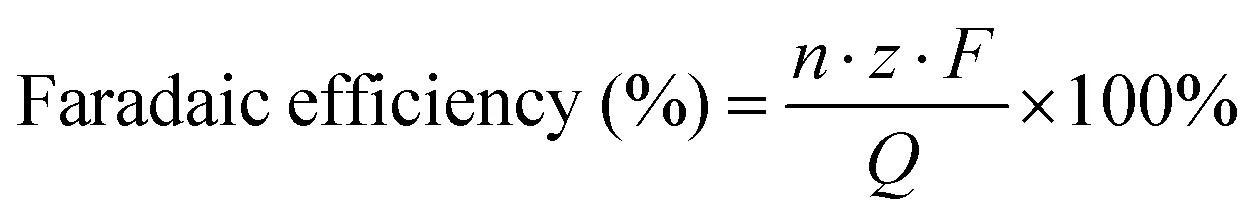
where *n* is the number of moles of product formed, calculated by multiplying the product concentration determined by HPLC by the volume of the remaining electrolyte solution, *z* is the number of electrons transferred per molecule of product formed (Table S2), *F* is Faraday's constant (96 485 C mol^−1^), and *Q* is the total charge passed during the application of 0.75 and 1.0 V_RHE_.8
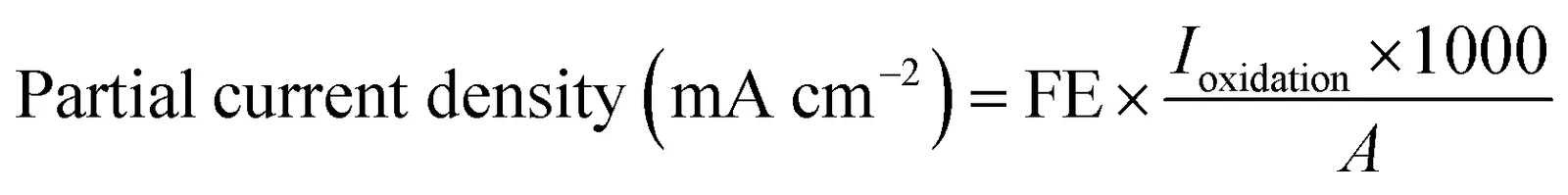
where *A* is the geometric surface area of the Pt RCE, and *I*_oxidation_ is from the charge passed during the 0.75 and 1.0 V_RHE_ steps, divided by the effective electrolysis time at these potentials. The effective electrolysis time was obtained by multiplying the total electrolysis duration by the fraction of the pulsing cycle spent at 0.75 and 1.0 V_RHE_. For example, for a pulsing protocol with *t*_0.75V_ = 0.5 s, *t*_1.0V_ = 0.5 s, *t*_0.3V_ = 1 s, the fraction of time at 0.75 and 1.0 V_RHE_ is 0.5, and the 6-hour electrolysis time was therefore multiplied by 0.5.

## Author contributions

K. P., C. G. M.-G., I. E. L. S., M. M. T., and A. H. conceived and designed the experiments. K. P. conducted experiments and analysed data. H. Y. assisted with HPLC measurements. K. P. and H. Y. wrote the manuscript with input from C. G. M.-G., I. E. L. S., M. M. T., and A. H. All the authors discussed the results and contributed to the final manuscript.

## Conflicts of interest

There are no conflicts of interest to declare.

## Supplementary Material

EY-004-D6EY00090H-s001

## Data Availability

The data supporting this article are included in the supplementary information (SI). Supplementary information: experimental details including platinum rotating cylinder electrode pretreatment and photographs of experimental setup, additional electrochemical results (CV and EIS measurements), HPLC calibration curves of all compounds, additional results of pulsed potential protocols, hydrodynamic analysis, Tafel analysis, regeneration test, and chemical stability test for glyceraldehyde. Ref. 60–66 are cited. See DOI: https://doi.org/10.1039/d6ey00090h. Numerical data is available at https://doi.org/10.5281/zenodo.21782115.
