# Supplementary material for: Coupling dynamic catalyst regeneration with convective transport enables stable and selective glycerol electrooxidation on platinum
Source: EES Catal. 2026 Aug 19;4(6):1412–23. doi: 10.1039/d6ey00090h (PMC13741091; doi:10.1039/d6ey00090h)
Supplement: Supplementary file 1 [file EY-004-D6EY00090H-s001.pdf]

Supplementary Information (SI) for EES Catalysis.
This journal is © The Royal Society of Chemistry 2026

Supplementary Information

Coupling dynamic catalyst regeneration with convective transport enables stable and selective glycerol electrooxidation on platinum

Kanyapat Plub-in,[a] Hanzhi Ye,[a] Carlos G. Morales-Guio,[b] Maria Magdalena Titirici,[ac] Ifan E.L. Stephens[d] and Anna Hankin*[a]

[a.] *Department of Chemical Engineering, Imperial College London, London, SW7 2AZ, UK. E-mail: anna.hankin@imperial.ac.uk*
[b.] *Department of Chemical and Biomolecular Engineering, University of California, Los Angeles, California, CA 90095, USA.*
[c.] *Advanced Institute for Materials Research (AIMR), Tohoku University 2-1-1 Katahira, Aoba-ku, Sendai 980-8577, Japan.*
[d.] *Department of Materials, Imperial College London, London, SW7 2AZ, UK.*

*Corresponding Author:

Anna Hankin (anna.hankin@imperial.ac.uk)

Experimental details

Pretreatment of Pt cylinder rotating electrode

A polycrystalline platinum cylinder electrode (Pt, length 8.0 mm, outer diameter 12 mm, Goodfellow, 99.95% metal basis) was used as the working electrode. Prior to all experiments, the Pt surface was mechanically polished using 1, 0.3, and 0.05 µm alumina suspensions on a fine polishing cloth (MicroCloth®) and then rinsed thoroughly with deionized water. To eliminate any impurities, the Pt cylinder electrode was heated using a blow torch with a propane/butane fuel gas (Propane & butane gas refill, RS PRO) for 1 min or until the entire surface turned bright orange and subsequently cooled to room temperature under an argon atmosphere,[60, 61] as shown in Fig. S1. After flame annealing, the Pt cylinder electrode was mounted on the 12 mm rotating cylinder electrode (RCE) tip (Metrohm Autolab). Once assembled, the electrode was transferred to the electrochemical cell and submerged in the electrolyte.

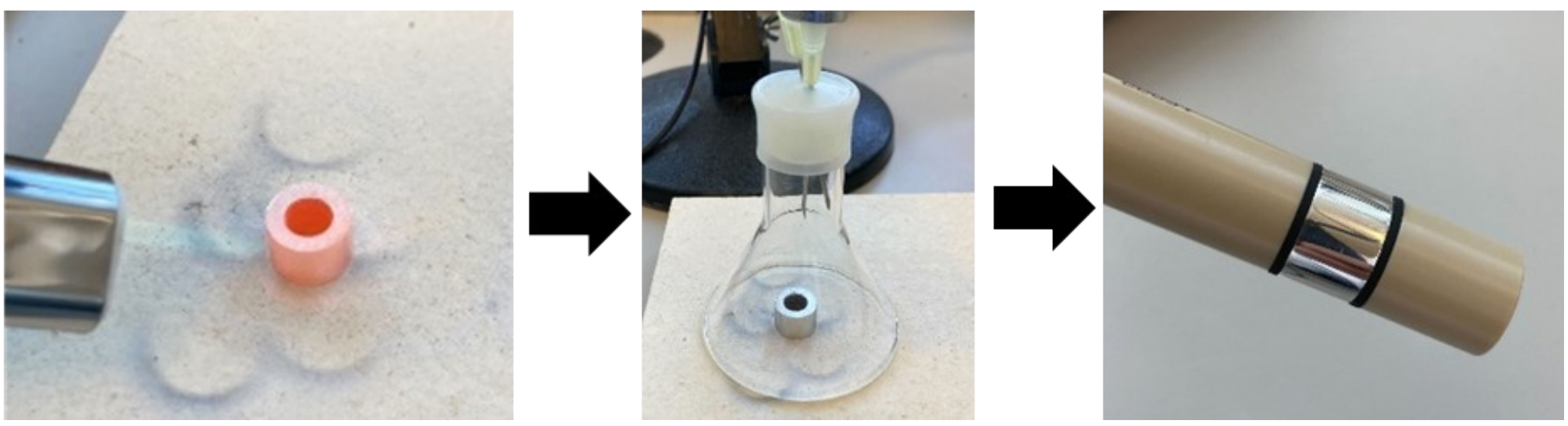

Fig. S1 Photographs of the flame annealing process for the Pt rotating cylinder electrode.

Electrochemical cell set up

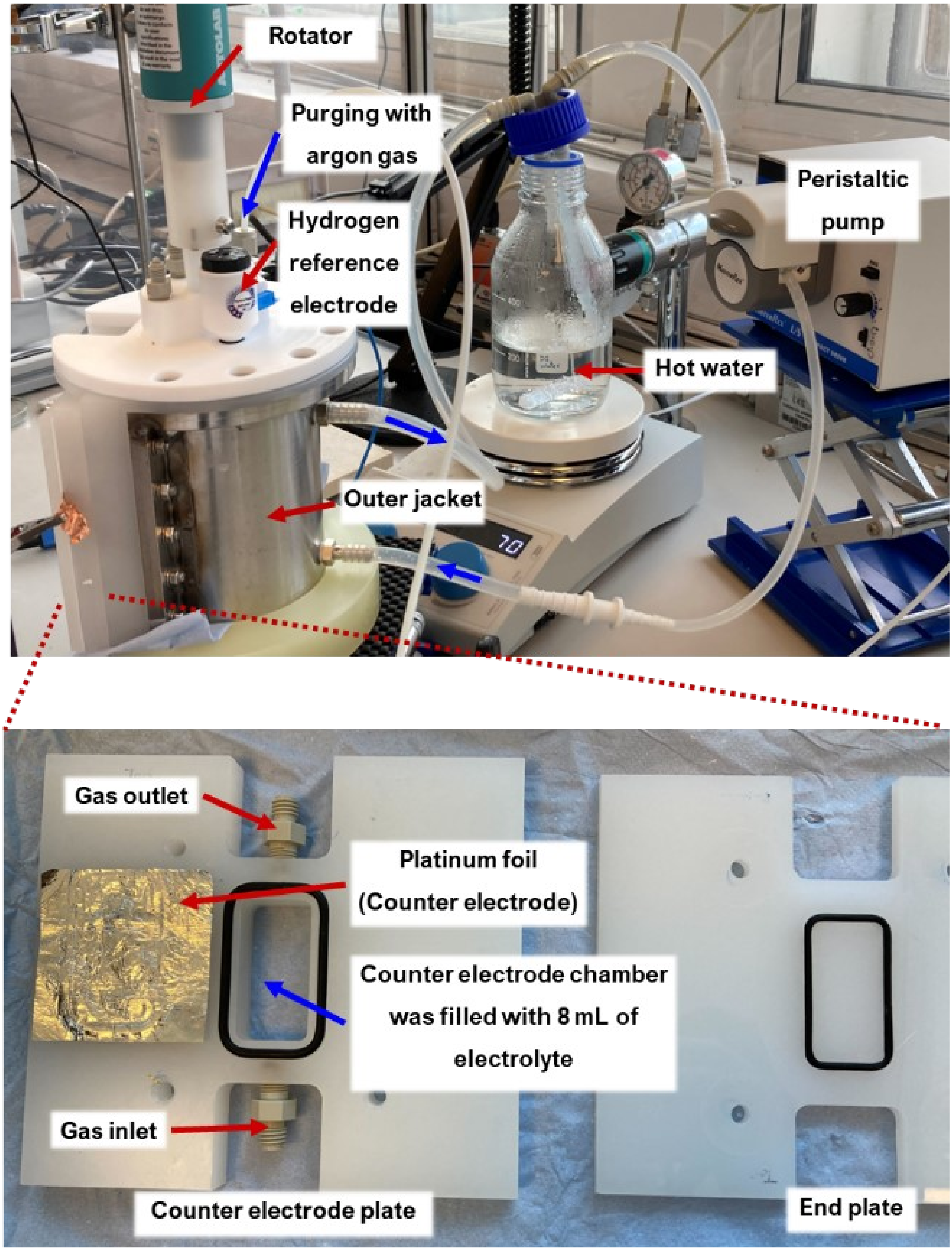

Fig. S2 Photographs of the experimental setup for GOR on Pt RCE.

Electrochemical measurements

We first examined the electrochemical surface area (ECSA) of the pretreated polycrystalline Pt RCE in 0.5 M H_2SO_4 at room temperature. After saturating the 0.5 M H_2SO_4 solution with argon gas, cyclic voltammetry with 10 cycles was performed at a scan rate of 50 mV s^{-1} and a rotation speed of 1000 rpm in the potential range of 0.03 to 1.4 V_{RHE}. The two oxidative peaks in the positive-going sweep were considered to be caused by the complete desorption of a hydrogen monolayer, initially present due to underpotential deposition (HUPD). To determine the total charge passed during HUPD, the area under the desorption peaks was calculated from 0.07 to 0.40 V_{RHE}, as shown in Fig. S3. The calculated charge was normalized by the geometric area of the electrode, i.e., an exposed surface area of Pt RCE ≈ 3.02 cm^2. The measured HUPD charge density of our clean, planar polycrystalline Pt RCE is consistent with the widely reported value of approximately 210 μC cm^{-2} for smooth polycrystalline Pt.[45] This agreement suggests that the electrochemically active surface area (ECSA) is approximately equal to the geometric surface area of the Pt RCE, reflecting the electrode's low surface roughness. Hence, the geometric surface area was used to normalize the measured current throughout this work.

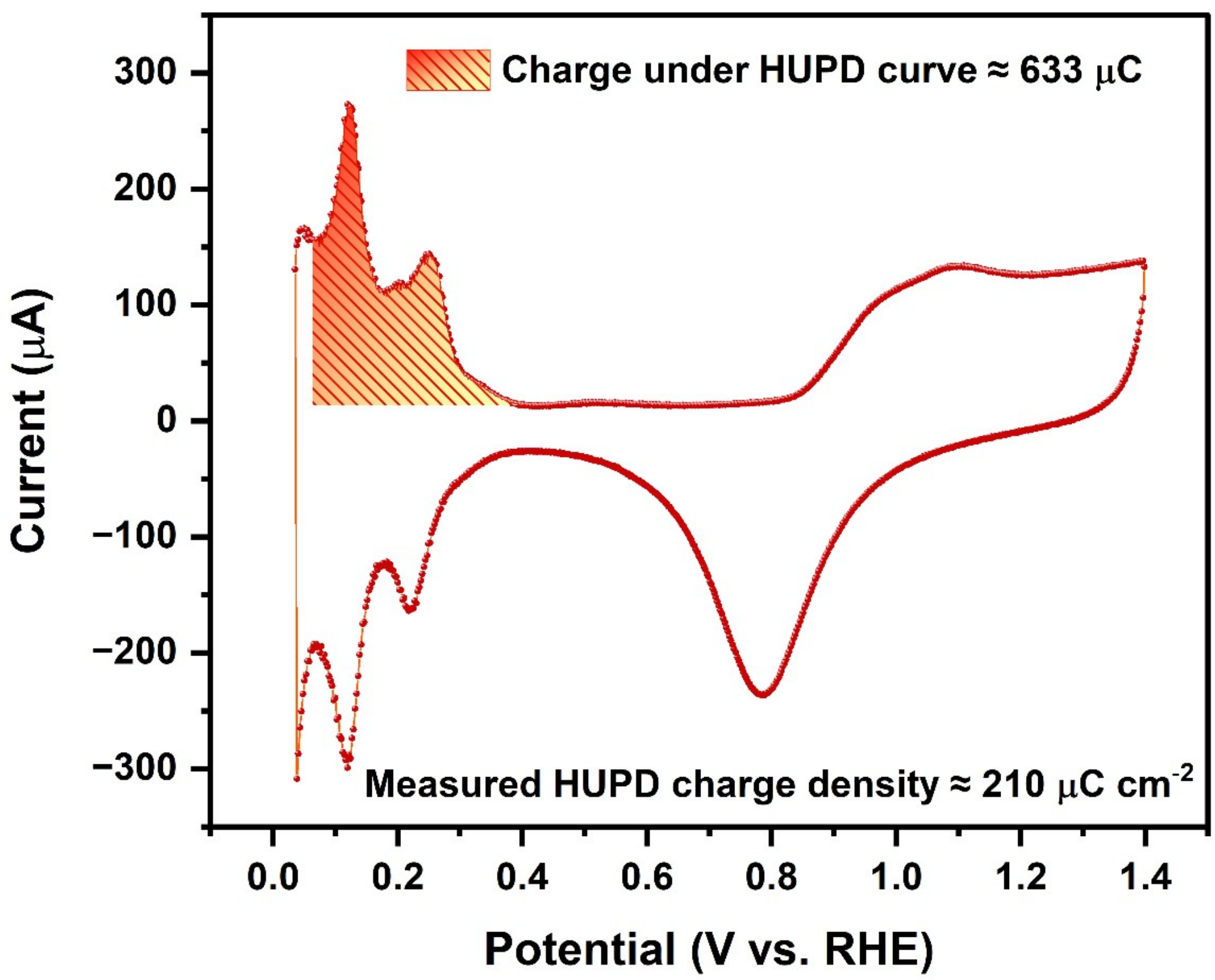

Fig. S3 Cyclic voltammogram of a polycrystalline Pt rotating cylinder electrode (RCE) in Ar-saturated 0.5 M H_2SO_4 recorded at room temperature with a scan rate of 50 mV s^{-1} and a rotation rate of 1000 rpm. The area under the desorption peaks was calculated from 0.07 to 0.40 V_{RHE}.

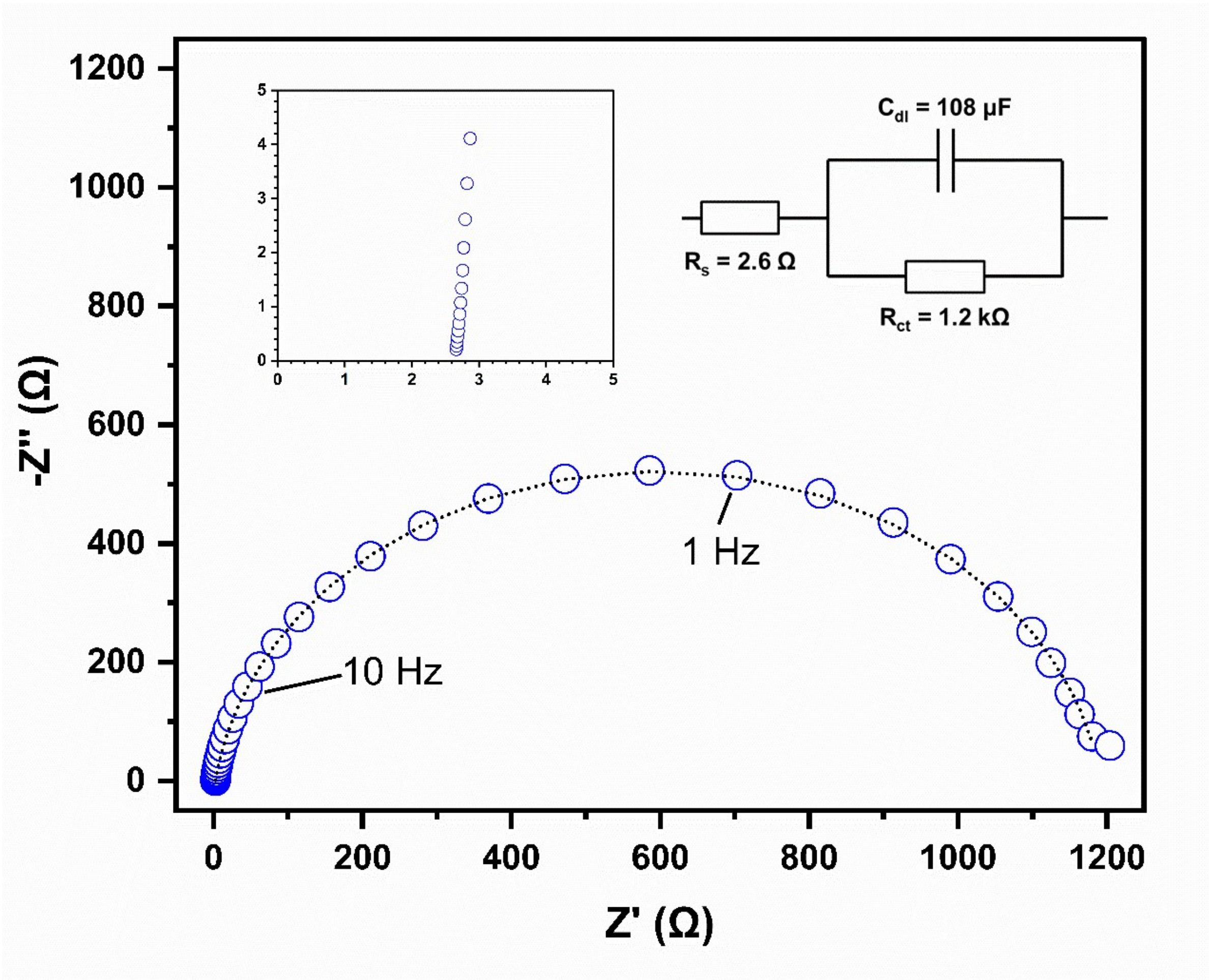

Fig. S4 Nyquist plot of the Pt RCE acquired at open circuit potential and the equivalent circuit used for quantifying the solution resistance.

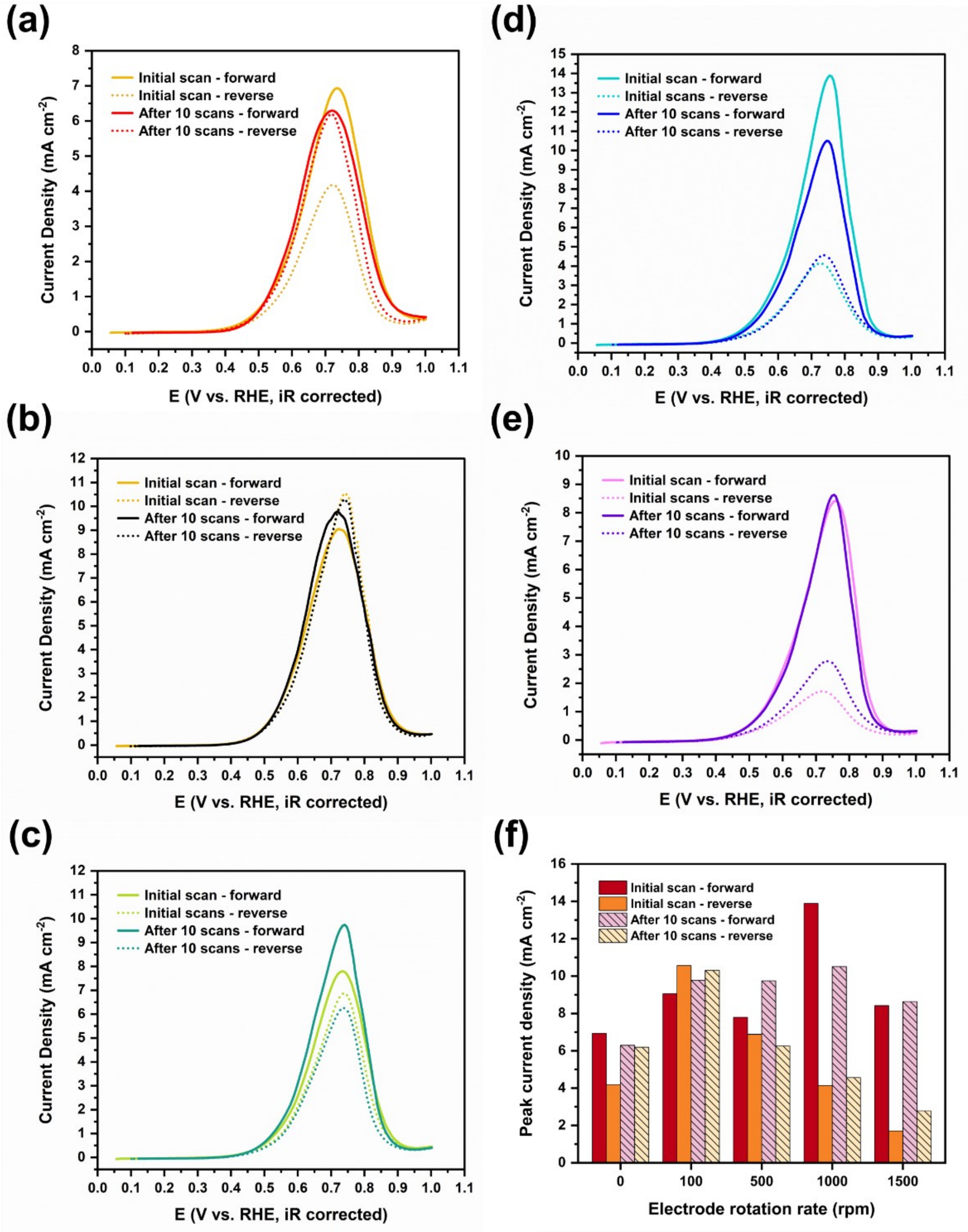

Fig. S5 The effect of electrode rotation on the CV of Pt RCE in 0.02 M glycerol and 0.1 M NaOH at 60 ºC, scan rate 50 mV s^{-1}, various rotation speeds: (a) 0 rpm, (b) 100 rpm, (c) 500 rpm, (d) 1000 rpm and (e) 1500 rpm. (f) The comparison of the peak current density at 0.75 V vs. RHE in a positive-going (forward) scan and a negative-going (reverse) scan of the CVs at different rotation speeds.

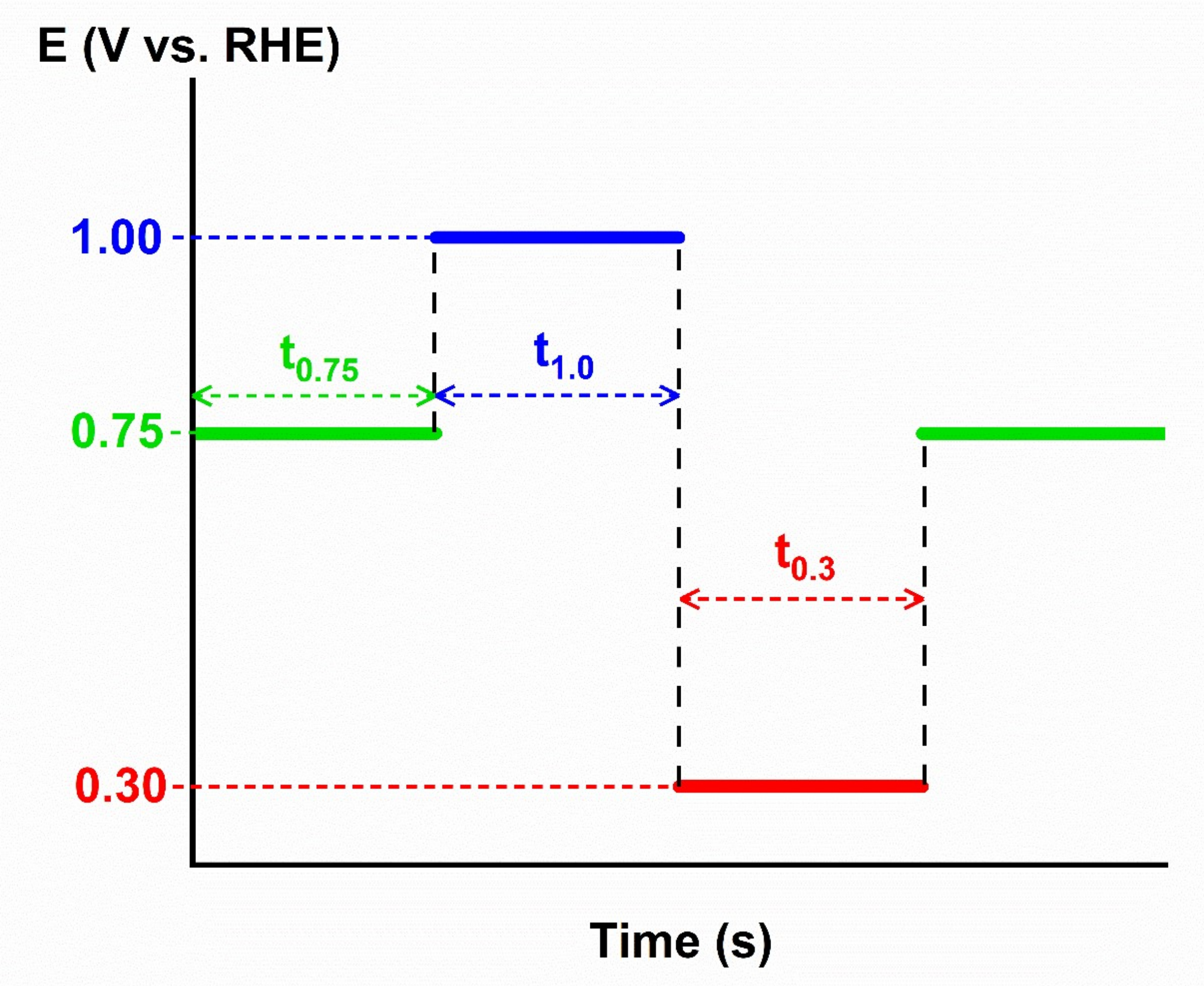

Fig. S6 Schematic of a three-step pulsed potential waveform

Table S1 Details of twenty-eight three-step pulsed potential protocols employed in this study.

Pulsing condition	Duration of each pulse potential (s)			Duty cycle of 0.75 V_{RHE} (%)	Total charge over 20 minutes (C)	Current density after 20 minutes (mA cm^{-2})
	$t_{0.75V}$	$t_{1.0V}$	$t_{0.3V}$			
P1	0.5	0.5	0.5	33.3	13.8	10.0
P2	0.5	0.5	1.0	25.0	10.6	10.5
P3	0.5	0.5	2.0	16.7	7.5	11.0
P4	0.5	1.0	0.5	25.0	11.4	10.3
P5	0.5	1.0	1.0	20.0	9.1	10.6
P6	0.5	1.0	2.0	14.3	6.9	11.0
P7	0.5	2.0	0.5	16.7	8.4	10.8
P8	0.5	2.0	1.0	14.3	7.7	11.3
P9	0.5	2.0	2.0	11.1	6.2	11.5
P10	1.0	0.5	0.5	50.0	14.0	6.9
P11	1.0	0.5	1.0	40.0	12.4	7.9
P12	1.0	0.5	2.0	28.6	9.70	8.7
P13	1.0	1.0	0.5	40.0	13.6	8.3
P14	1.0	1.0	1.0	33.3	12.2	9.1
P15	1.0	1.0	2.0	25.0	9.6	9.5
P16	1.0	2.0	0.5	28.6	10.2	8.3
P17	1.0	2.0	1.0	25.0	9.2	8.7
P18	1.0	2.0	2.0	20.0	8.1	9.8
P19	2.0	0.5	0.5	66.7	16.4	5.9
P20	2.0	0.5	1.0	57.1	13.8	5.9
P21	2.0	0.5	2.0	44.4	10.9	6.3
P22	2.0	1.0	0.5	57.1	12.9	5.6
P23	2.0	1.0	1.0	50.0	12.5	6.2
P24	2.0	1.0	2.0	40.0	11.1	7.4
P25	2.0	2.0	0.5	44.4	11.1	5.7
P26	2.0	2.0	1.0	40.0	11.2	7.0
P27	2.0	2.0	2.0	33.3	10.1	7.6
P28	4.0	0.5	0.5	80.0	13.5	4.0

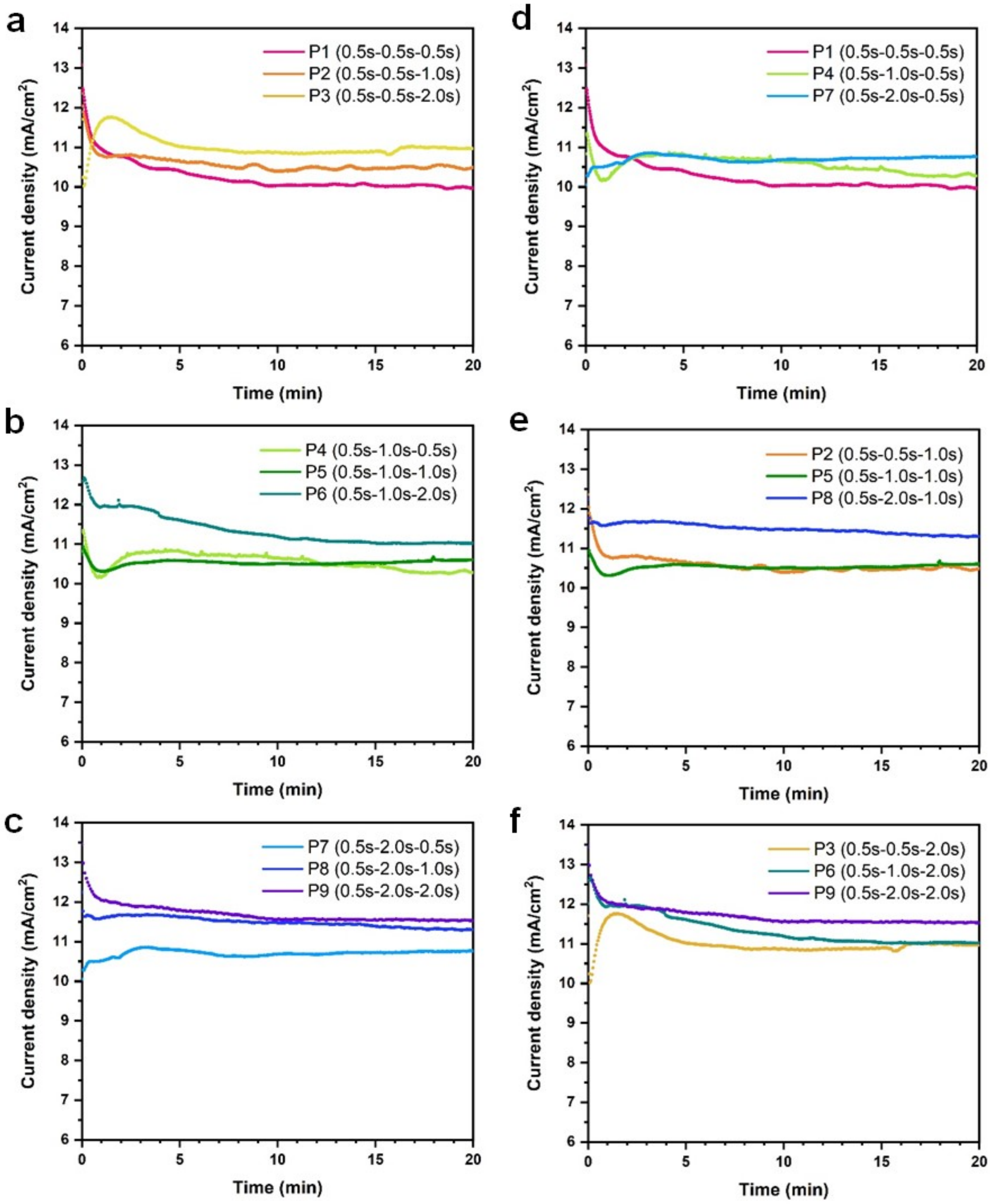

Fig. S7 Comparison of average current densities during 0.75 V_{RHE} obtained under three-step pulsed potential protocols with a glycerol oxidation step duration $t_{0.75V}$ of 0.5 s (P1 – P9). (a)-(c) show comparisons when $t_{1.0V}$ is fixed. (d)-(f) show comparisons when $t_{0.3V}$ is fixed.

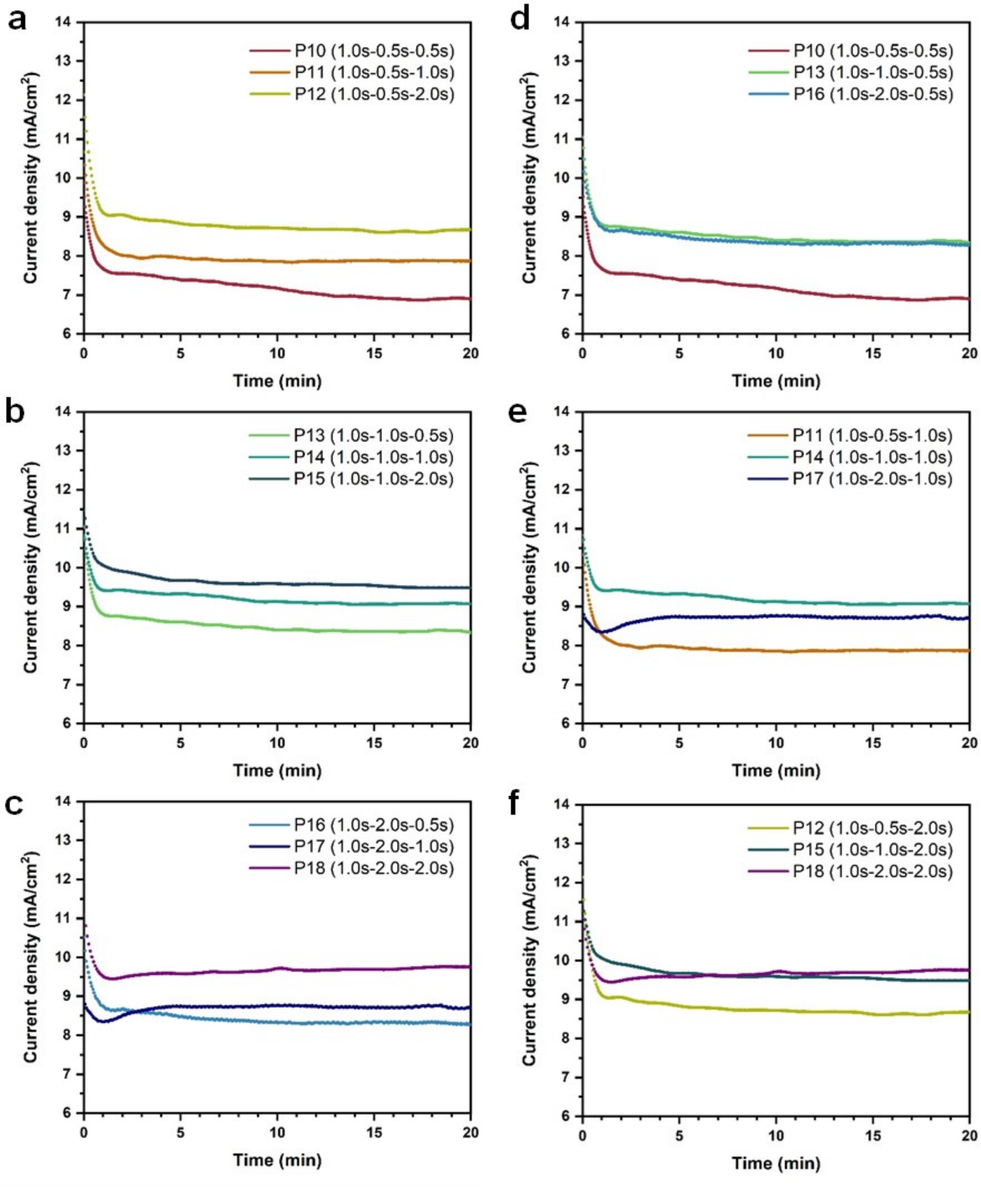

Fig. S8 Comparison of average current densities during 0.75 V_{RHE} obtained under three-step pulsed potential protocols with a glycerol oxidation step duration $t_{0.75V}$ of 1 s (P10 – P18). (a)-(c) show comparisons when $t_{1.0V}$ is fixed. (d)-(f) show comparisons when $t_{0.3V}$ is fixed.

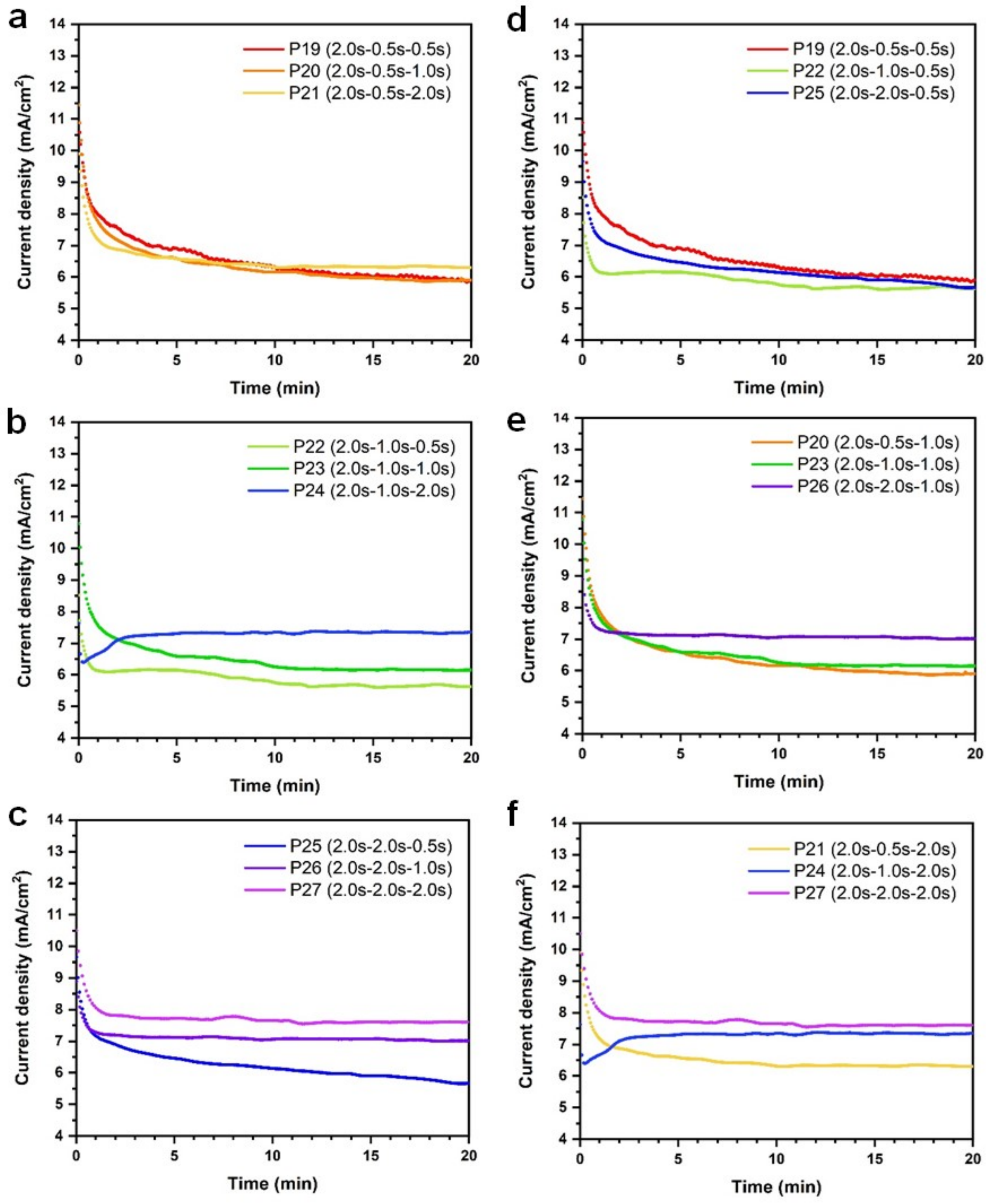

Fig. S9 Comparison of average current densities during 0.75 V_{RHE} obtained under three-step pulsed potential protocols with a glycerol oxidation step duration $t_{0.75V}$ of 2 s (P19 – P27). (a)-(c) show comparisons when $t_{1.0V}$ is fixed. (d)-(f) show comparisons when $t_{0.3V}$ is fixed.

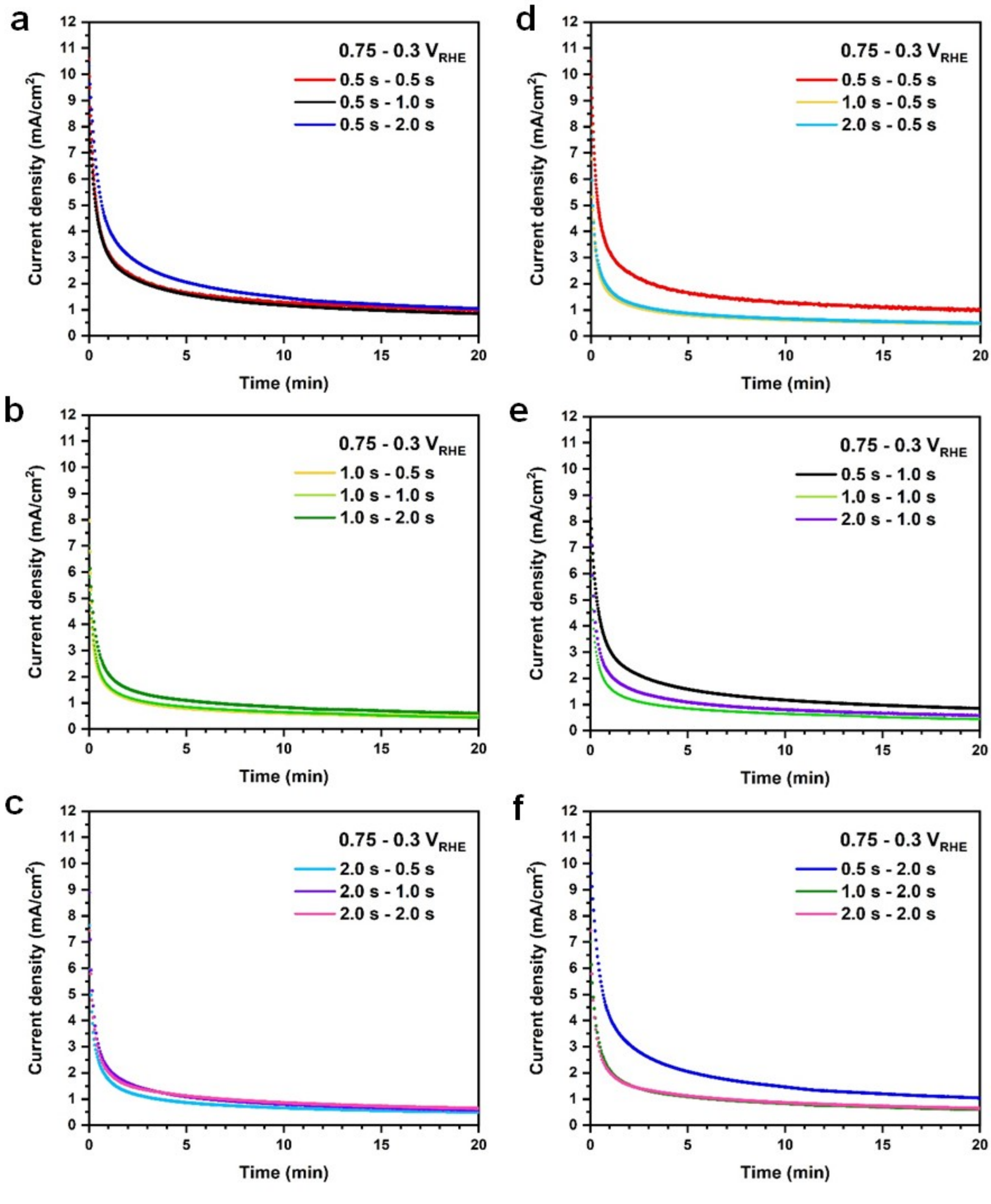

Fig. S10 Comparison of average current densities during 0.75 V_{RHE} obtained under two-step pulsed potential protocols at 0.75 V_{RHE} and 0.3 V_{RHE}.

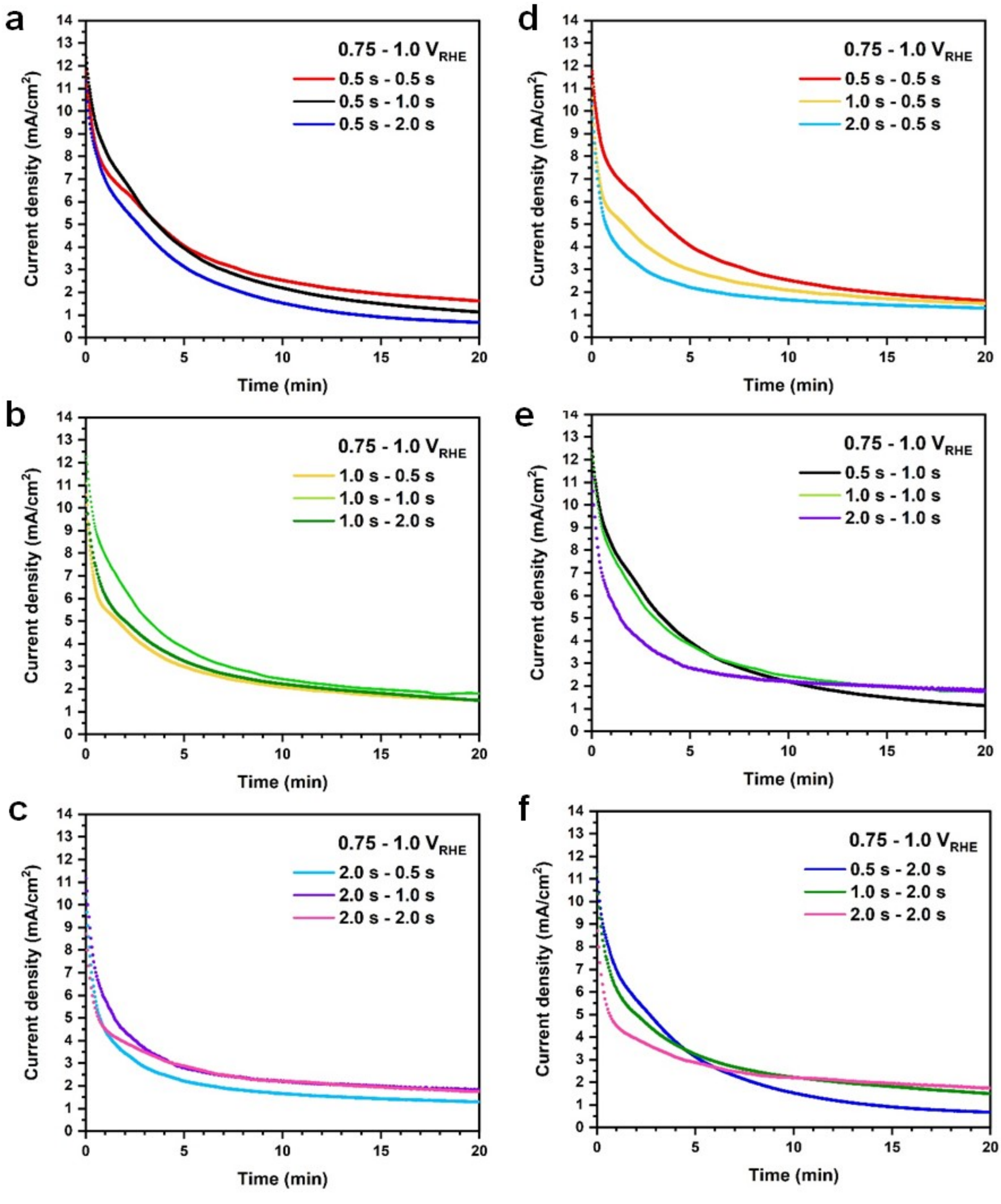

Fig. S11 Comparison of average current densities during 0.75 V_{RHE} obtained under two-step pulsed potential protocols at 0.75 V_{RHE} and 1.0 V_{RHE}.

Table S2 Summary of the number of electrons transferred per molecule of product formed during glycerol electrooxidation.

**Products**	**no. electron transferred**
Glycerate (GLA)	4
Lactate (LA)	2
Tartronate (TA)	8
Oxalate (OA)	22/3
Glyoxylate (GOA)	16/3
Glycolate (GCA)	10/3
Formate (FA)	8/3

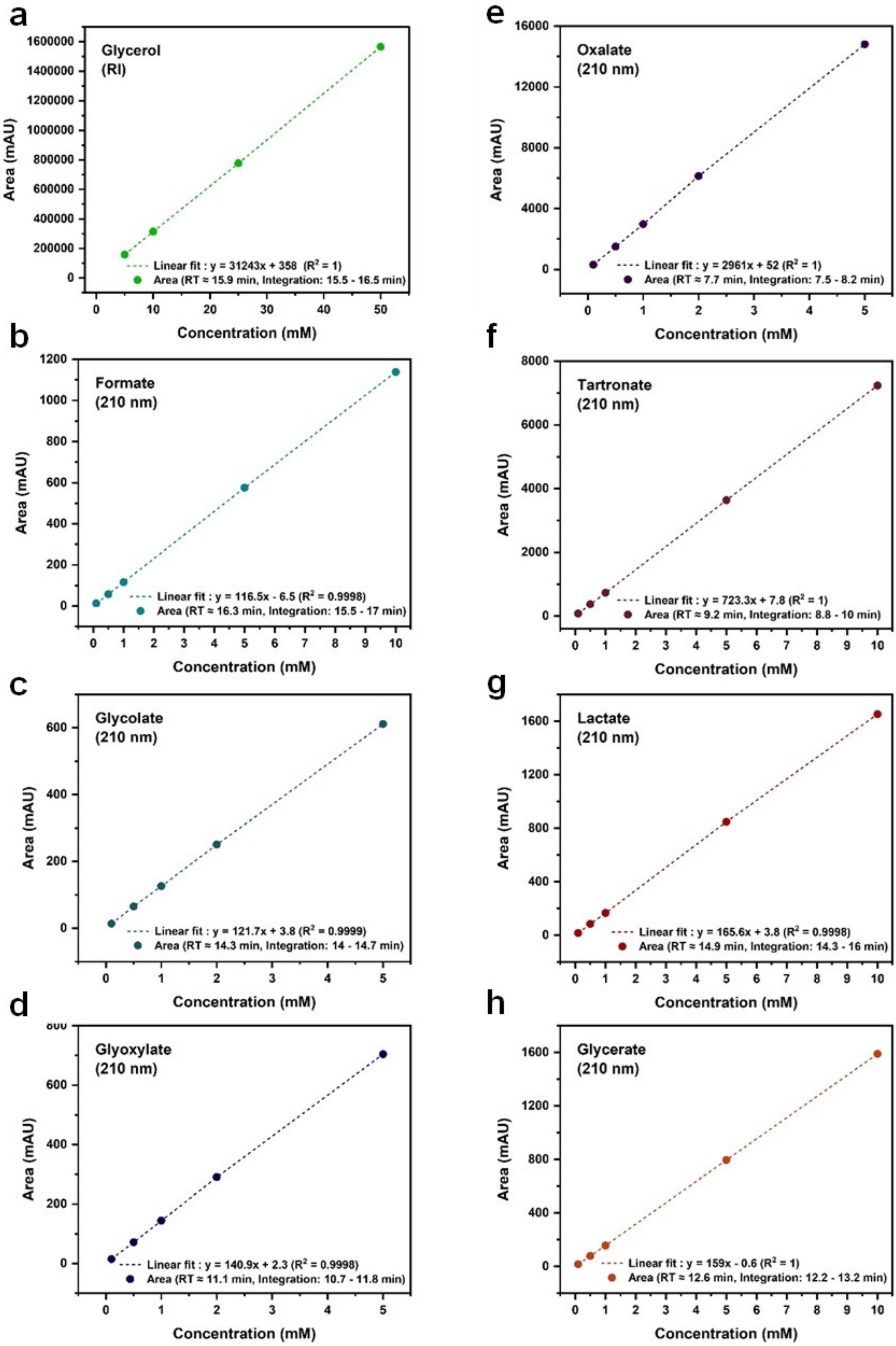

Fig. S12 HPLC calibration curves of (a) glycerol, (b) formate, (c) glycolate, (d) glyoxylate, (e) oxalate, (f) tartronate, (g) lactate, (h) glycerate.

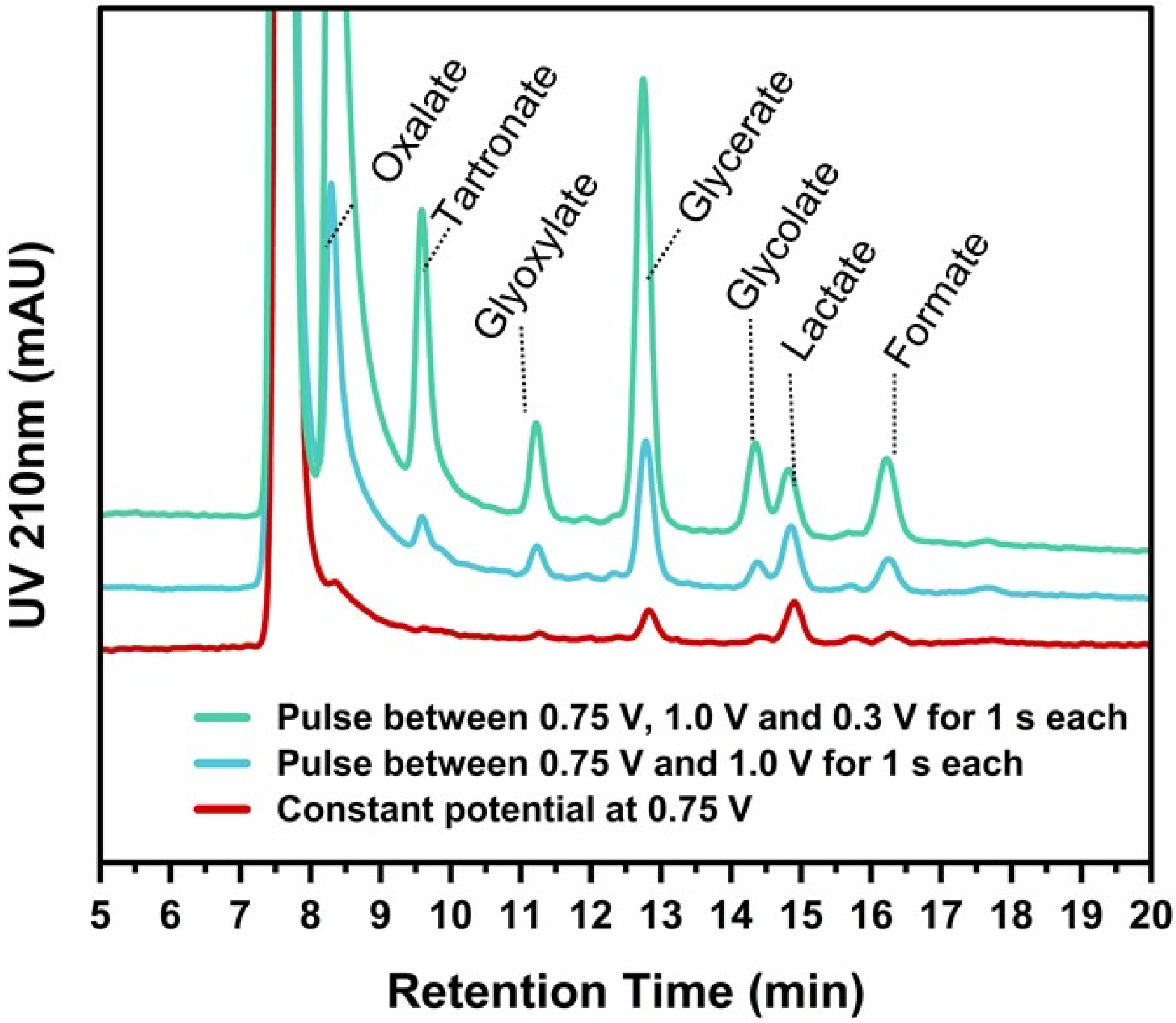

Fig. S13 Comparison of HPLC chromatograms with collected samples from 2-hour constant potential electrolysis, two-step, and three-step pulsed potential electrolysis. The peak at ca. 7.2 minutes is the dissolved Na_2SO_4 from the dilution and acidification.

Additional results

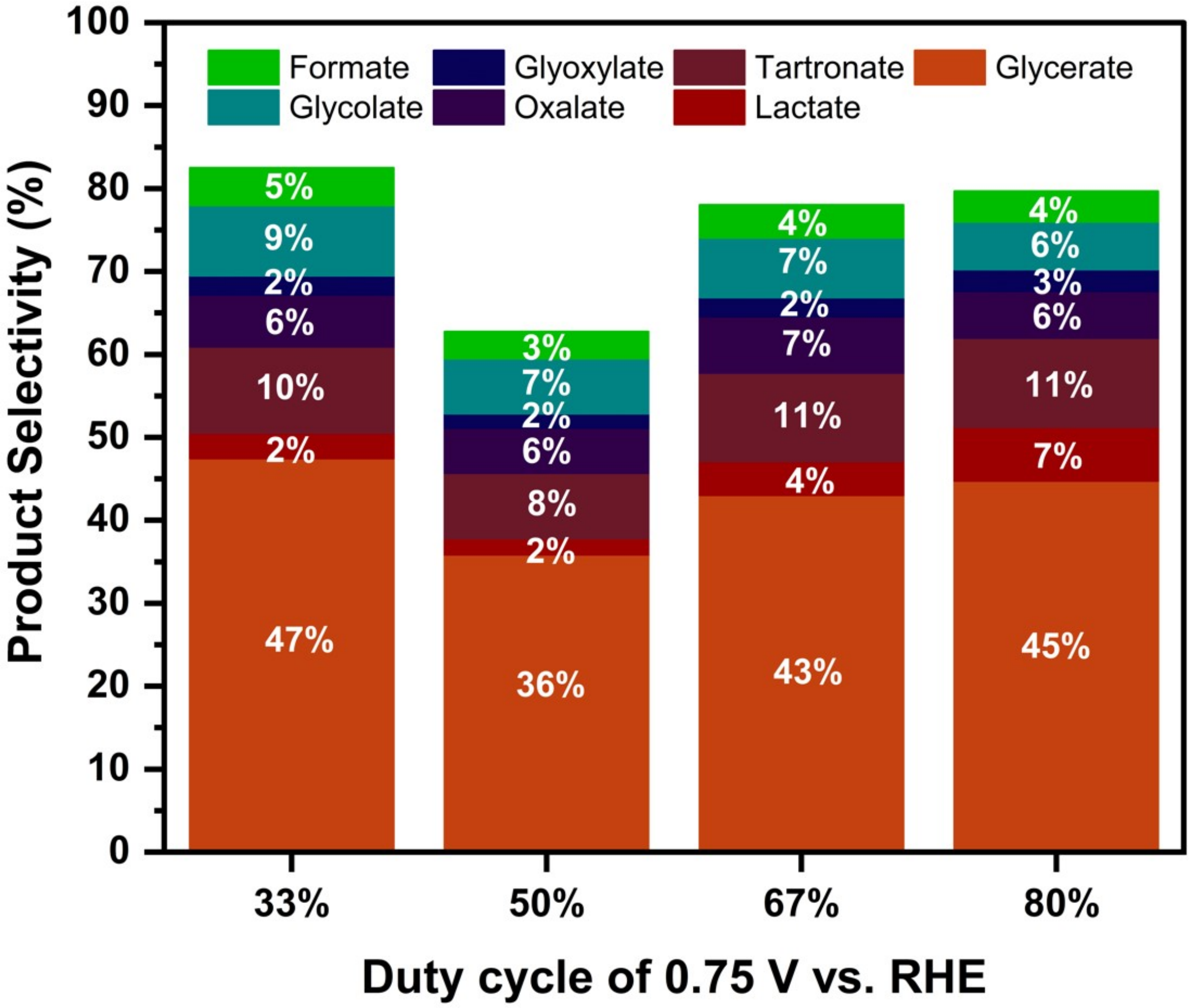

Fig. S14 Product selectivity after 6-hour glycerol electrolysis using three-step pulsed potential protocols with varying duty cycles. The duration of the glycerol oxidation step ($t_{0.75V}$) was set to 0.5, 1.0, 2.0, and 4.0 s, while $t_{1.0V}$ and $t_{0.3V}$ were fixed at 0.5 s, in 0.02 M glycerol and 0.1 M NaOH at 60 °C under static conditions (0 rpm)

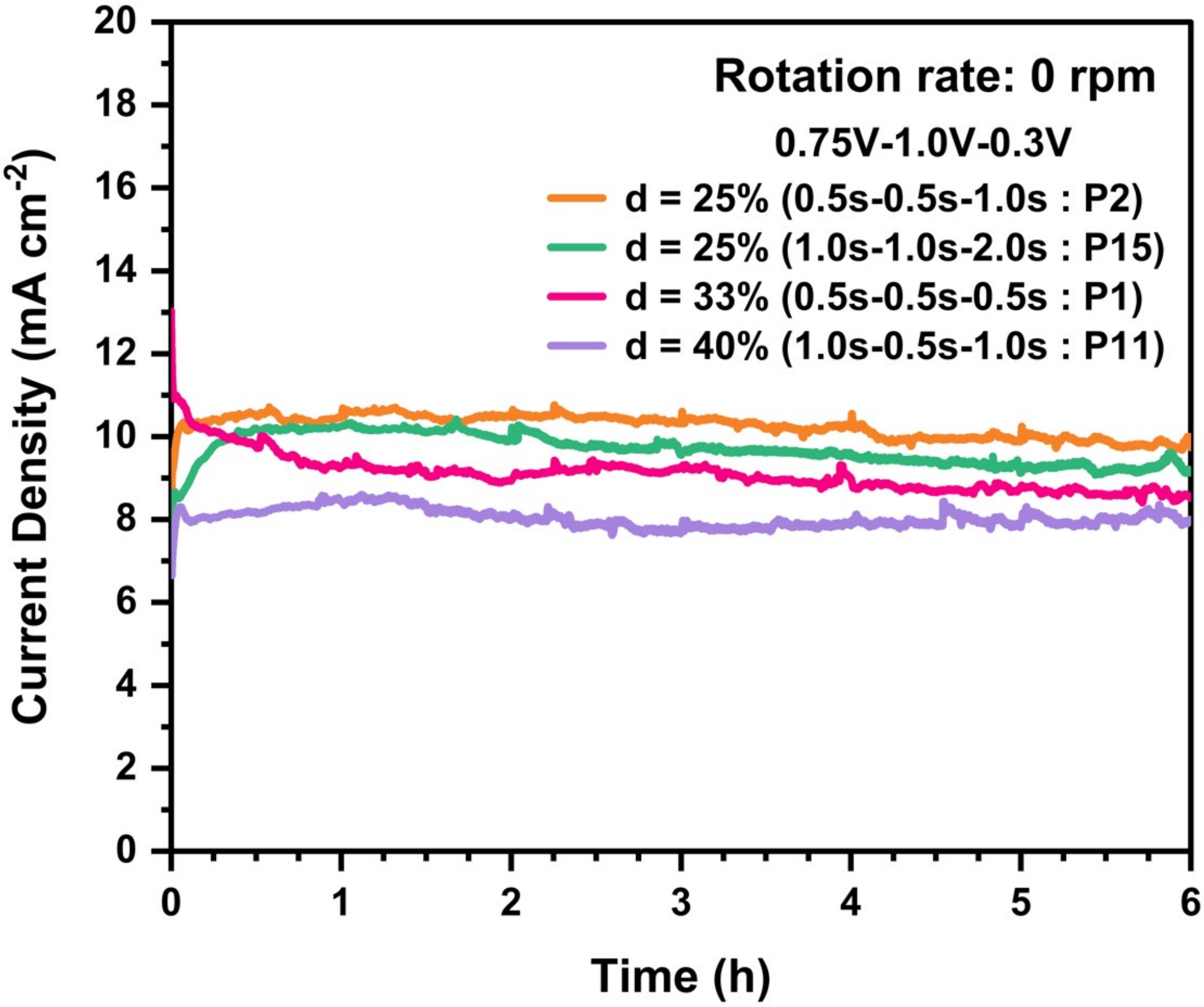

Fig. S15 Average current density comparison after 6-hour glycerol electrolysis using three-step pulsed potential protocols with varying duty cycles in 0.02 M glycerol and 0.1 M NaOH at 60 °C under static conditions (0 rpm) : (yellow) 25% (P2), (pink) 33.3% (P1), (green) 25% (P15), and (purple) 40% (P11).

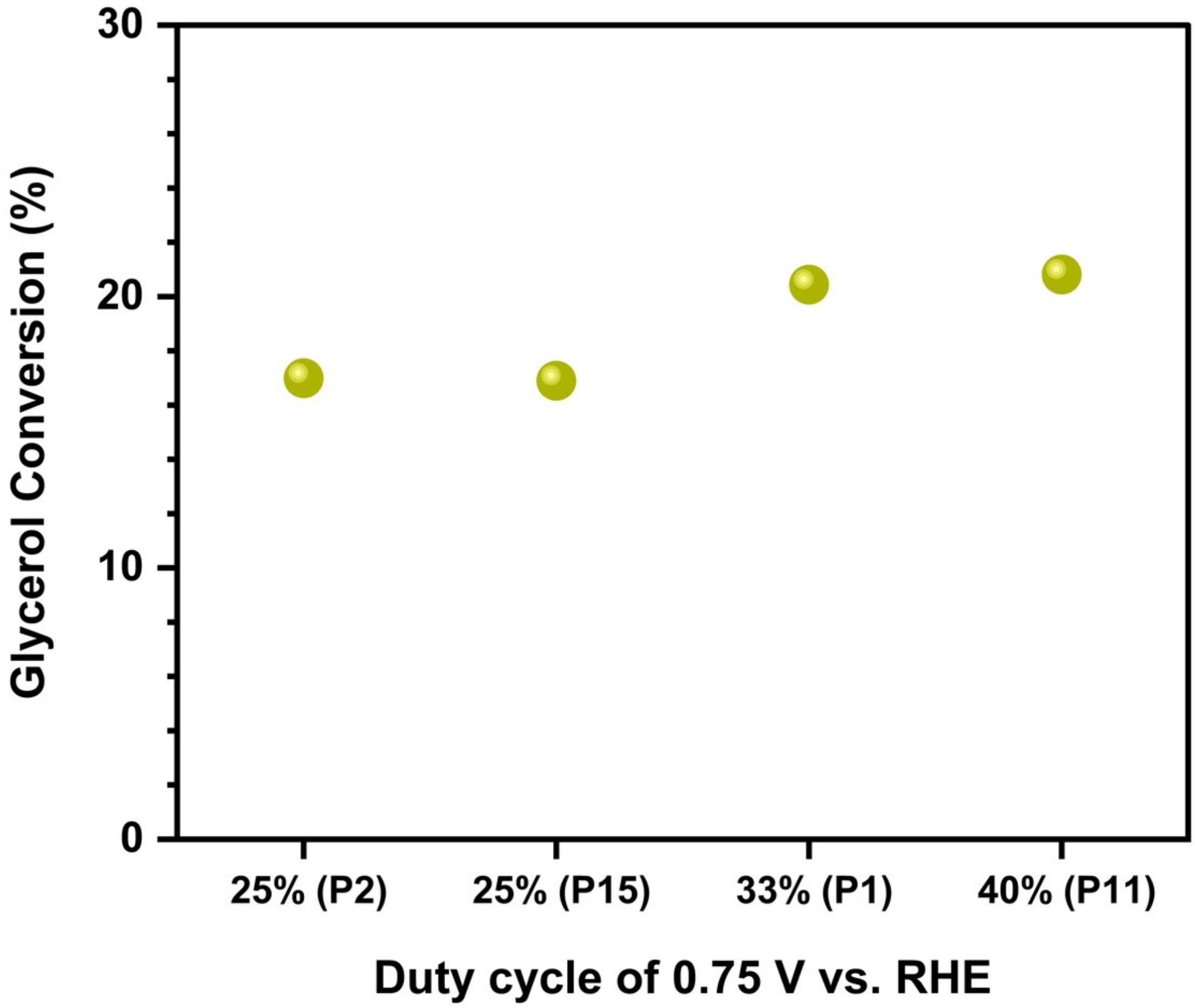

Fig. S16 Glycerol conversion comparison after 6-hour glycerol electrolysis using three-step pulsed potential protocols with varying duty cycles in 0.02 M glycerol and 0.1 M NaOH at 60 °C under static conditions (0 rpm) : 25% (P2), 33.3% (P1), 25% (P15), and 40% (P11).

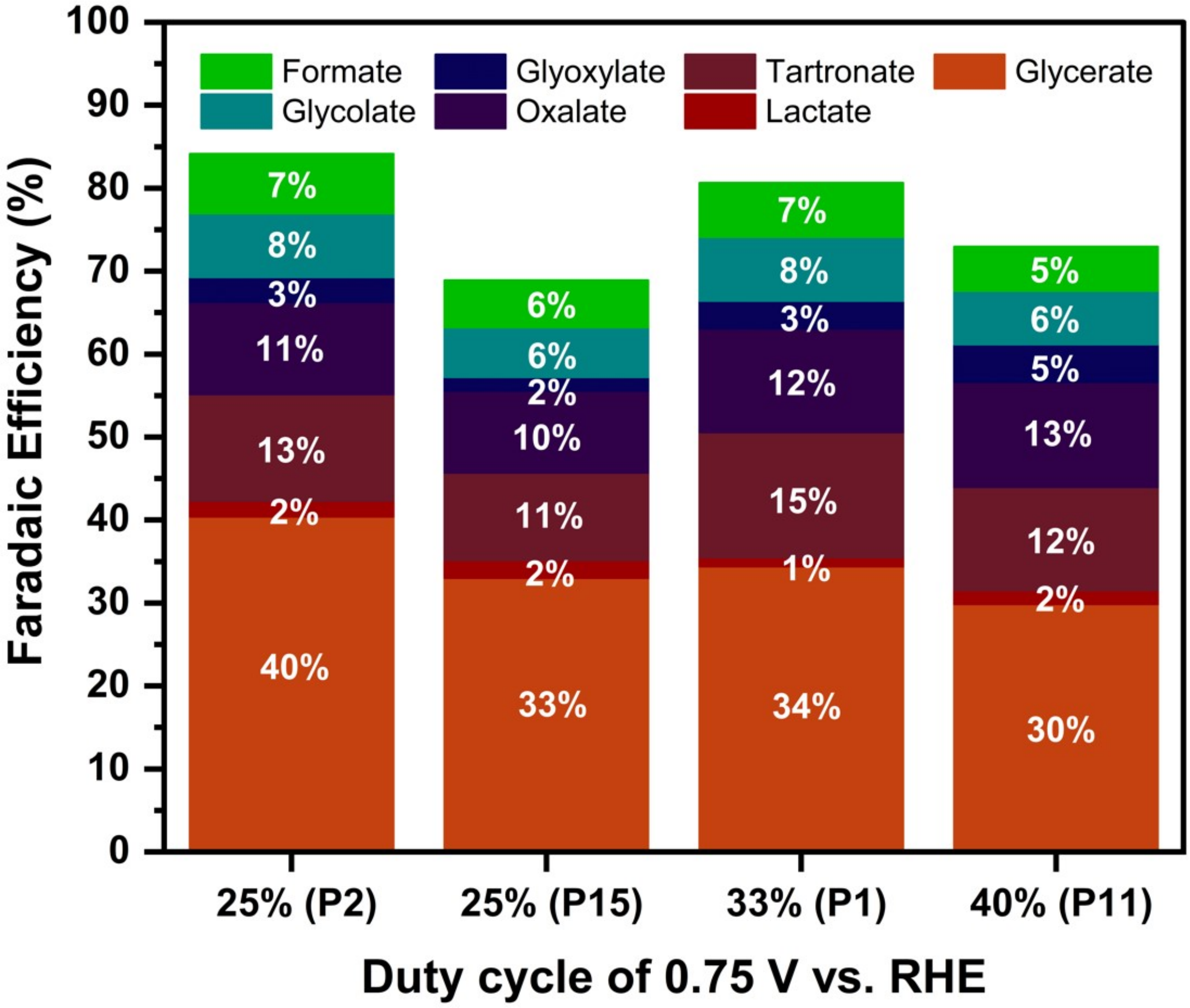

Fig. S17 Faradaic efficiency comparison after 6-hour glycerol electrolysis using three-step pulsed potential protocols with varying duty cycles in 0.02 M glycerol and 0.1 M NaOH at 60 °C under static conditions (0 rpm) : 25% (P2), 33.3% (P1), 25% (P15), and 40% (P11).

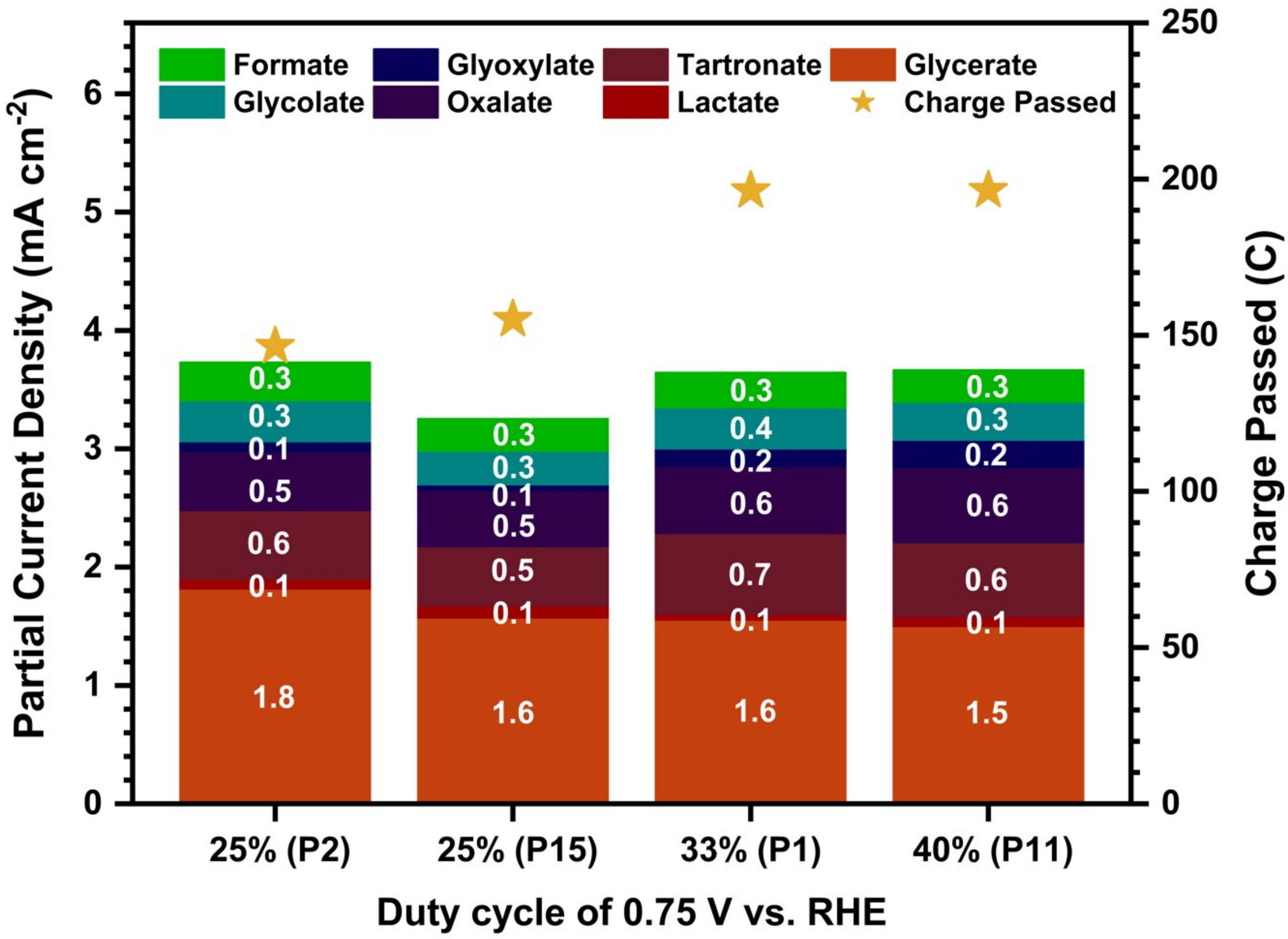

Fig. S18 Comparisons of partial current density and charge passed after 6-hour glycerol electrolysis using three-step pulsed potential protocols with varying duty cycles in 0.02 M glycerol and 0.1 M NaOH at 60 °C under static conditions (0 rpm) : 25% (P2), 33.3% (P1), 25% (P15), and 40% (P11).

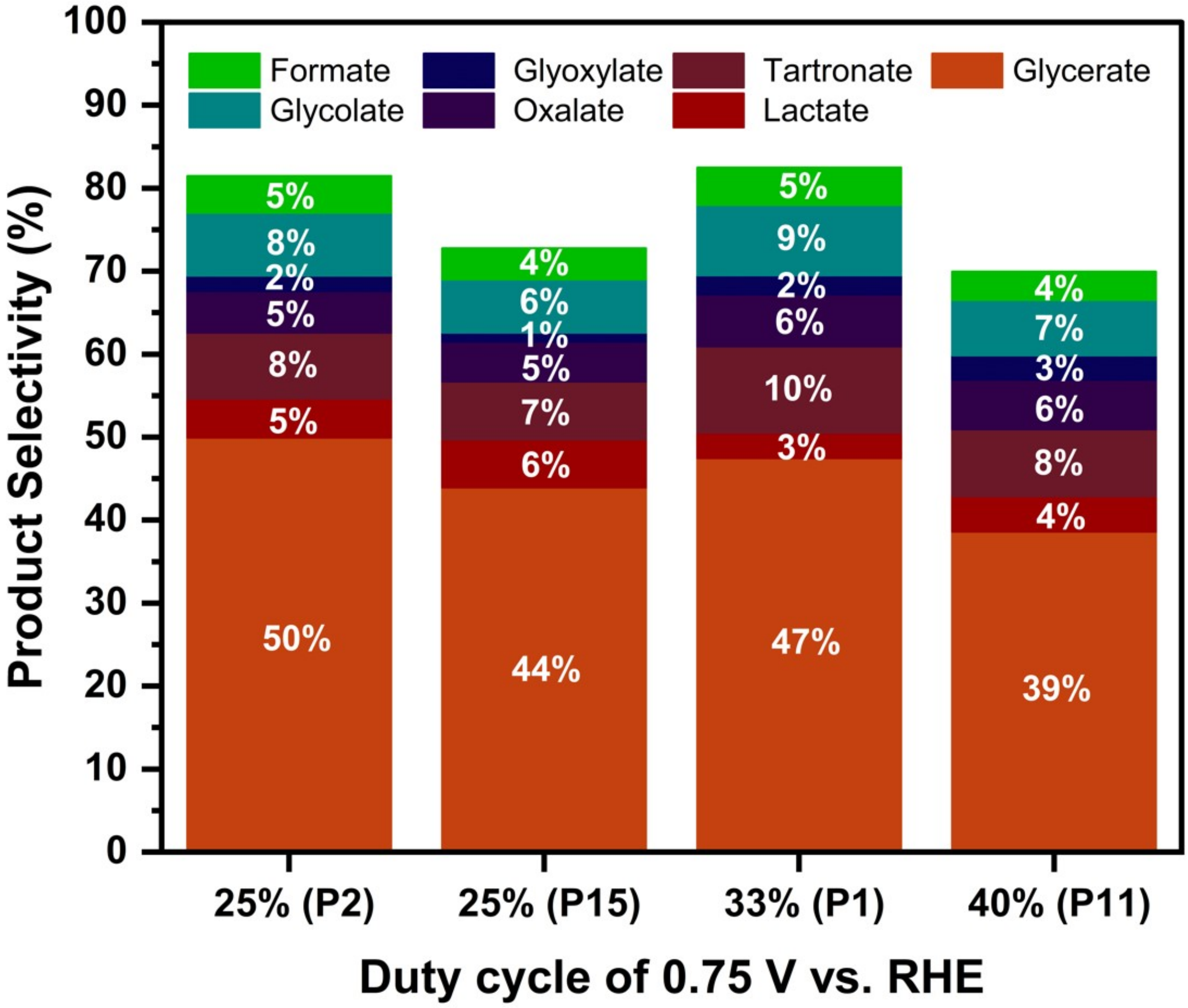

Fig. S19 Product selectivity comparison after 6-hour glycerol electrolysis using three-step pulsed potential protocols with varying duty cycles in 0.02 M glycerol and 0.1 M NaOH at 60 °C under static conditions (0 rpm) : 25% (P2), 33.3% (P1), 25% (P15), and 40% (P11).

Hydrodynamic analysis

To estimate the mass-transfer coefficients (k_m) for GOR in the RCE system, a dimensionless correlation developed by Eisenberg et al. for rotating cylinder electrodes under turbulent flow conditions was applied.[62, 63] The correlation between the Sherwood number (Sh), Reynolds number (Re), and Schmidt number (Sc) is given by Supplementary Equation (S1):

$$Sh = 0.0791 \cdot Re^{0.7} \cdot Sc^{0.356} \tag{S1}$$

The Sherwood number describes the relationship between the mass-transfer coefficient (k_m), the molecular diffusion coefficient of glycerol (D), and the characteristic length of the system, which is the cylinder diameter (d_{cyl}) in this case:

$$Sh = \frac{k_m \cdot d_{cyl}}{D} \tag{S2}$$

The Reynolds number was calculated based on the peripheral velocity of the cylinder surface (U_{cyl}) and the kinematic viscosity (ν) of the electrolyte solution:

$$Re = \frac{U_{cyl} \cdot d_{cyl}}{\nu} \tag{S3}$$

where the peripheral velocity of the rotating cylinder electrode is related to the rotation frequency (f, rpm) and cylinder diameter as follows:

$$U_{cyl} = \frac{\pi \cdot f \cdot d_{cyl}}{60} \tag{S4}$$

The Schmidt number, which represents the ratio between momentum diffusivity and mass diffusivity, was calculated using:

$$Sc = \frac{\nu}{D}$$

(S5)

The glycerol diffusion coefficient in water at 25 °C was obtained from literature values, and the diffusion coefficient at the operating temperature of 60 °C was estimated to approximately double due to the reduced viscosity and enhanced molecular mobility. Thus, the glycerol diffusion coefficient was assumed to be $D \approx 2.00 \times 10^{-9}$ m^2 s^{-1}.[64, 65] The kinematic viscosity of the electrolyte solution (0.02 M glycerol in 0.1 M NaOH) was assumed to be comparable to that of water at 60 °C and was estimated as $\nu \approx 4.74 \times 10^{-7}$ m^2 s^{-1}.[66]

The calculated mass-transfer coefficients were then used to estimate the corresponding limiting current densities (j_L) based on Faraday's law:

$$j_L = \frac{z \cdot F \cdot k_m \cdot C_b}{A}$$

(S6)

where *z* represents the number of electrons transferred per molecule of product formed, which was assumed to be 4 based on the major oxidation product identified in this study, glycerate, F is Faraday's constant (96485 C mol^{-1}), C_b is the bulk concentration of glycerol (mol m^{-3}), and *A* is the geometric surface area of the Pt RCE.

The calculated mass-transfer coefficients and the corresponding mass transport-limiting current densities are summarized in Table S3, together with the experimental current densities obtained from the CV curves shown in Fig. S5 and three-step pulsed potential measurements. As the electrode rotation rate increases, the measured current densities evaluated at the applied potentials corresponding to the CV peaks (0.75 V_{RHE}) diverge considerably from the calculated mass transport-limiting current densities. This shows that the operation in both the constant potential and three-step pulsed potential protocols is not limited by mass transport.

Table S3 Details of the calculation of mass-transfer coefficients (k_m) and corresponding limiting current densities (j_L) compared to experimental current densities obtained from CV results and three-step pulsed potential results.

Rotation frequenc y (f, rpm)	*Re*	*Sh*	Estimated mass-transfer coefficient (k_m, m s^{-1})	Estimated limiting current density (j_L, mA cm^{-2})	Measured current density at 0.75 V_{RHE} from CV results (mA cm^{-2})	Measured current density at 0.75 V_{RHE} from three-step pulsed potential results (mA cm^{-2})
100	1591	96.5	1.61×10^{-5}	12.4	9.2	13.9
500	7953	298	4.96×10^{-5}	38.3	9.7	13.6
1000	15907	483	8.06×10^{-5}	62.2	10.5	12.6
1500	23860	643	1.07×10^{-4}	82.7	8.6	11.7

Tafel analysis

To evaluate the reaction kinetics under different electrode rotation speeds, Tafel slopes were determined from the CV curves shown in Fig. S5. An example of the Tafel plot obtained from CV data without electrode rotation (0 rpm) is presented in Fig. S20. As summarized in Table S4, the Tafel slopes increased with increasing rotation speed, which can be attributed to the accelerated accumulation of poisoning intermediates on the active sites. Electrochemical glycerol oxidation is a complex multistep, multielectron-transfer process involving: (1) diffusion of glycerol from the bulk electrolyte to the catalyst surface; (2) adsorption of glycerol on the catalyst surface; (3) catalytic oxidation of glycerol by a surface-active oxygen species; (4) desorption of oxidation products from the catalyst surface; and (5) diffusion of products back into the electrolyte.[17] With increasing rotation speed, enhanced convective transport rapidly supplies fresh glycerol molecules to the electrode surface, thereby accelerating the initial oxidation step. However, the faster reaction rate also promotes the generation and accumulation of strongly adsorbed intermediates, leading to increased surface poisoning and catalyst deactivation.

Table S4 Summary of the Tafel slope calculated from CV data at different rotation speeds.

Electrode rotation speed (RPM)	Tafel Slope ($mV\ dec^{-1}$)
0	88
100	96
500	100
1000	103
1500	116

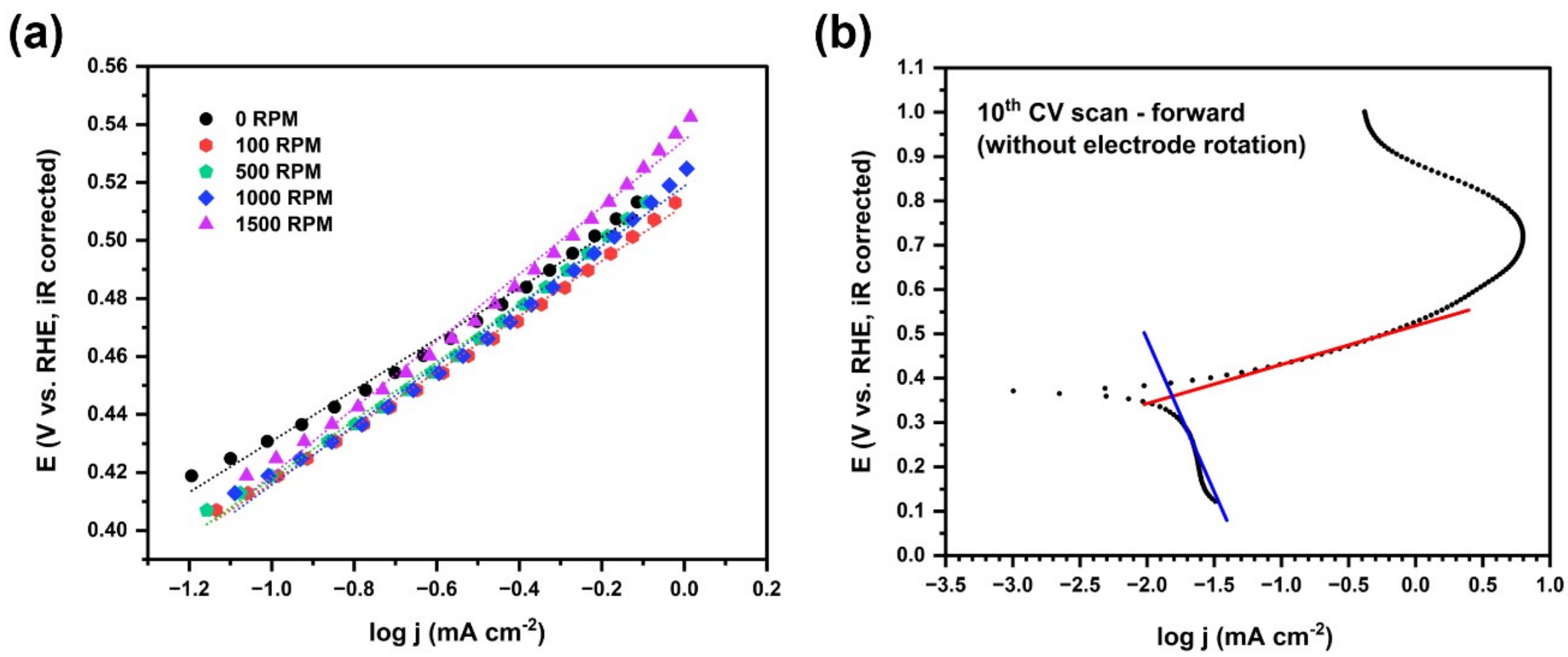

Fig. S20 (a) Tafel plots for GOR performance at different rotation speeds (b) Example of Tafel analysis of the GOR derived from CV data obtained using Pt RCE in 0.02 M glycerol and 0.1 M NaOH at 60 °C, recorded at a scan rate of 50 mV s^{-1} and a rotation speed of 0 rpm. The red and blue lines represent the anodic and cathodic Tafel fittings, respectively.

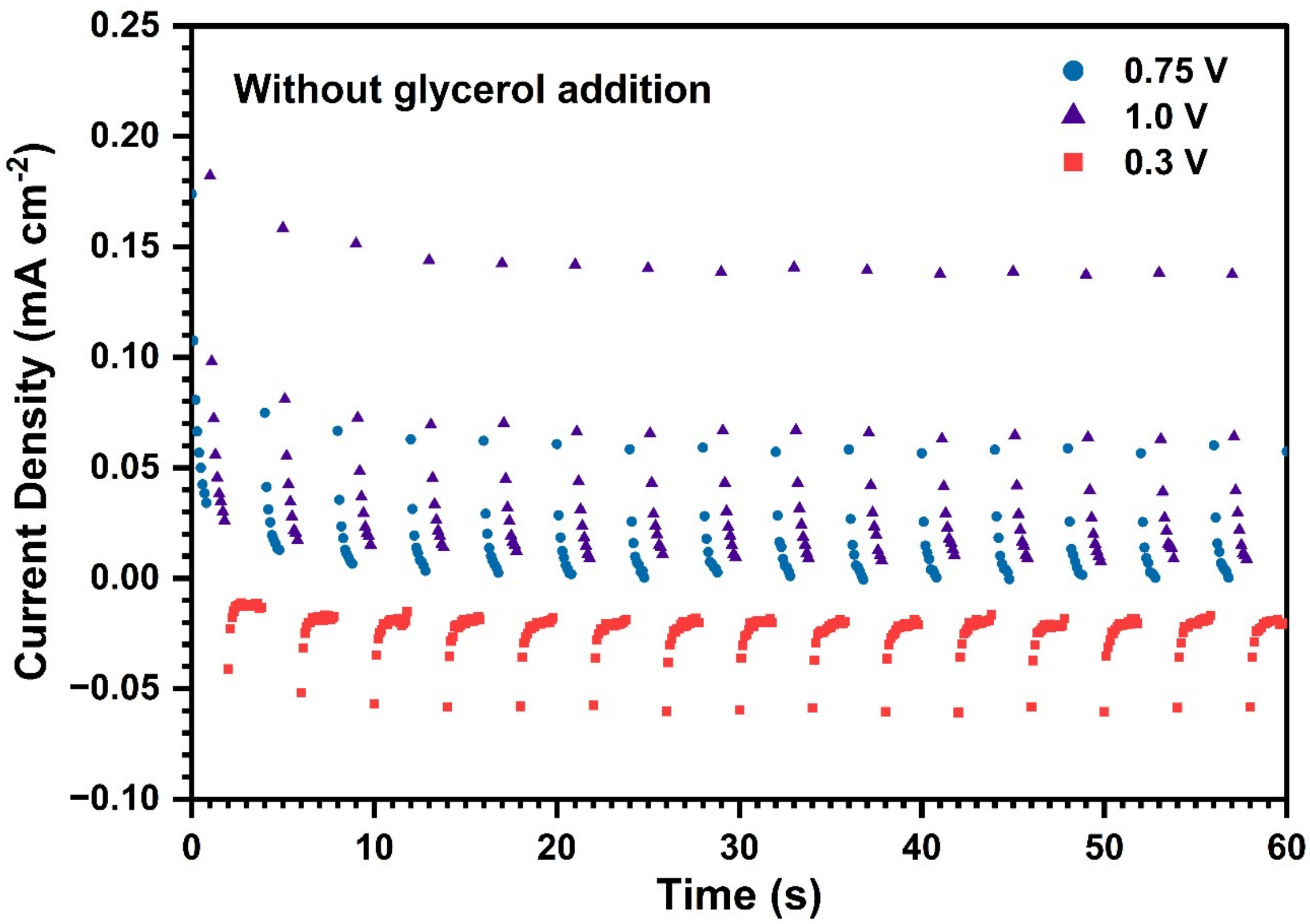

Fig. S21 Current density responses obtained using the three-step pulsed potential protocol P15 ($t_{0.75V}$ = 1 s, $t_{1.0V}$ = 1 s, $t_{0.75V}$ = 2 s) in 0.1 M NaOH at 60 °C under static conditions (0 rpm).

Regeneration Test

To further investigate whether catalyst surface reactivation occurs after severe catalyst deactivation, we applied the P15 pulsing condition after a constant-potential electrolysis experiment at 0.75 V_{RHE}, during which the current density decayed to nearly zero after 2 h, indicating substantial surface deactivation (see Fig. S22). The three-step potential pulsing was then applied for 3 min without removing the electrode from the electrolyte (0.02 M glycerol and 0.1 M NaOH). The separate current responses at each pulse potential are shown in Fig. S23 a–c, while the combined current response during the first 45 s is presented in Fig. S23 d. In Fig. S23 a, the current associated with the GOR at 0.75 V_{RHE} was gradually restored, whereas the current response at 0.3 V_{RHE} (Fig. S23 b, d) exhibits the underlying surface regeneration process. At the beginning of 0.3 V_{RHE} pulse, the capacitive current gradually changed from negative to positive as the GOR current at 0.75 V_{RHE} increased. This transition suggests progressive cleaning of the catalyst surface and the regeneration of accessible active sites, enabling enhanced adsorption and deprotonation of glycerol. Therefore, the increased faradaic contribution from the GOR process resulted in the observed shift of the capacitive current toward positive values.

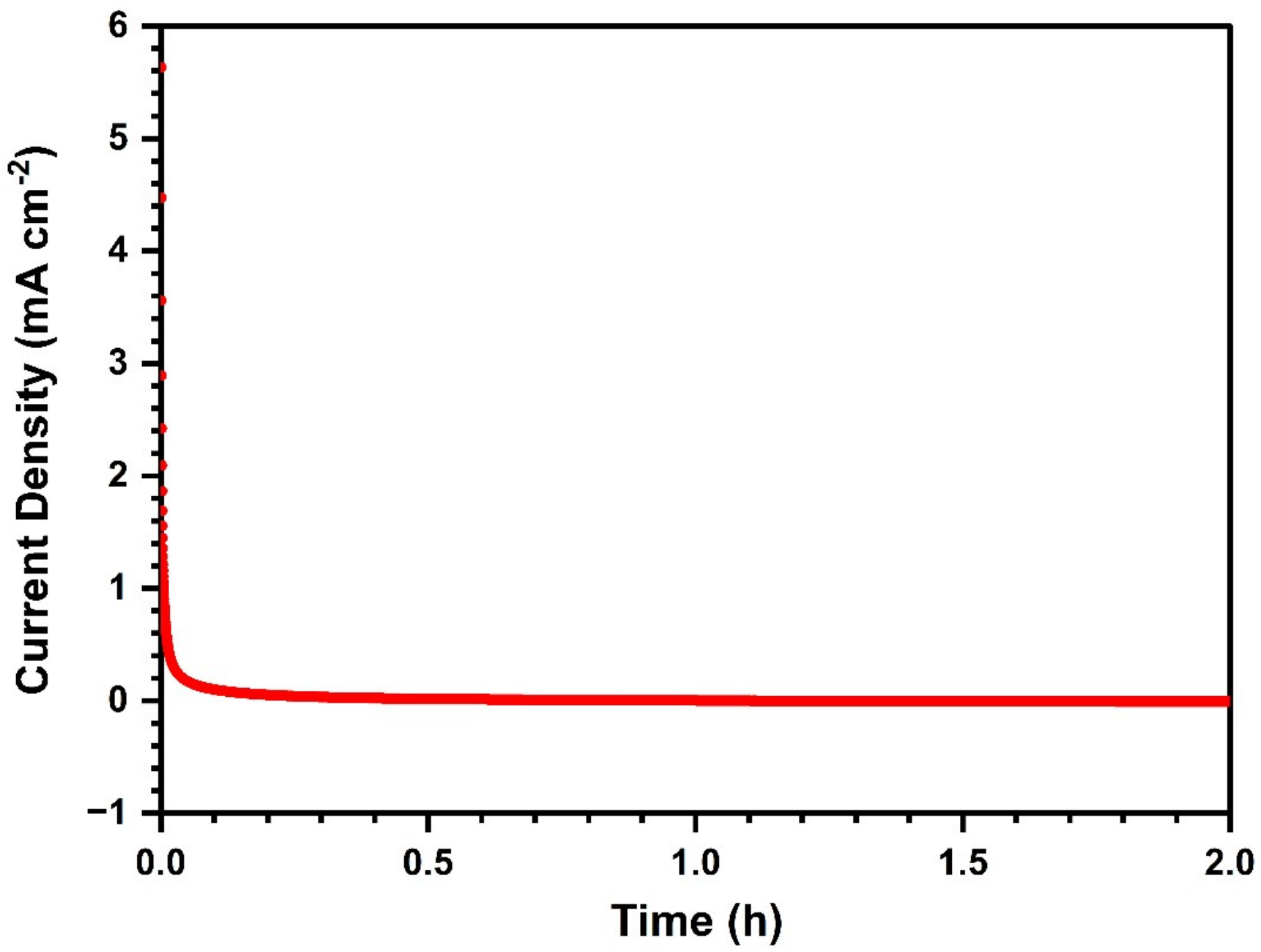

Fig. S22 Current density as a function of time obtained using constant potential electrolysis at 0.75 V_{RHE} in 0.02 M glycerol and 0.1 M NaOH at 60 °C and a rotation rate of 1500 rpm.

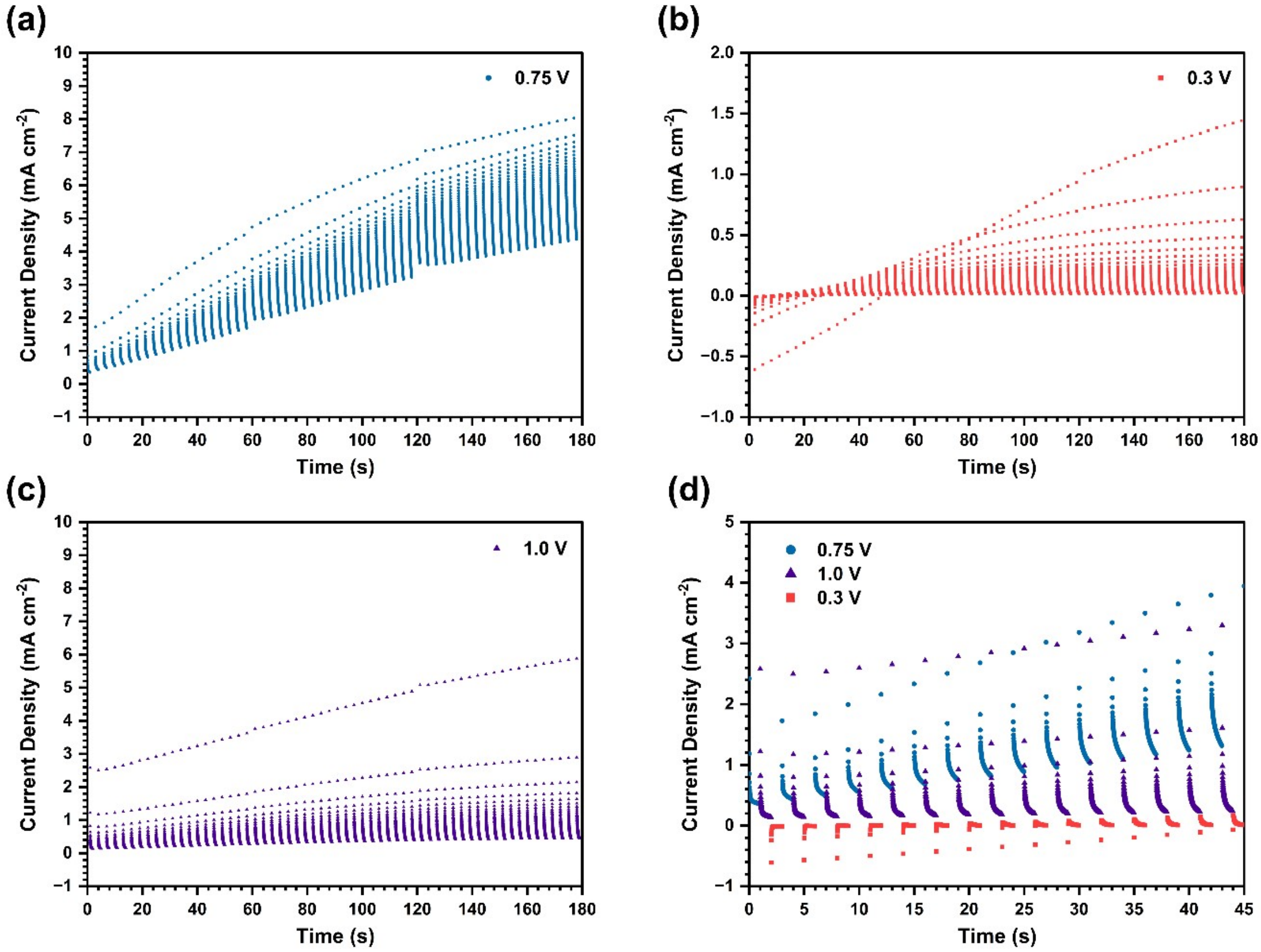

Fig. S23 Current density responses obtained using the three-step pulsed potential protocol P15 ($t_{0.75V}$ = 1 s, $t_{1.0V}$ = 1 s, $t_{0.75V}$ = 2 s) in 0.02 M glycerol and 0.1 M NaOH at 60 °C under static conditions (0 rpm): current density response at (a) 0.75 V_{RHE}, (b) at 0.30 V_{RHE}, (c) at 1.0 V_{RHE}, and (d) combined current density response with an enlarged view of the first 45 s. The current signal was recorded every 0.01 s.

Glyceraldehyde Test

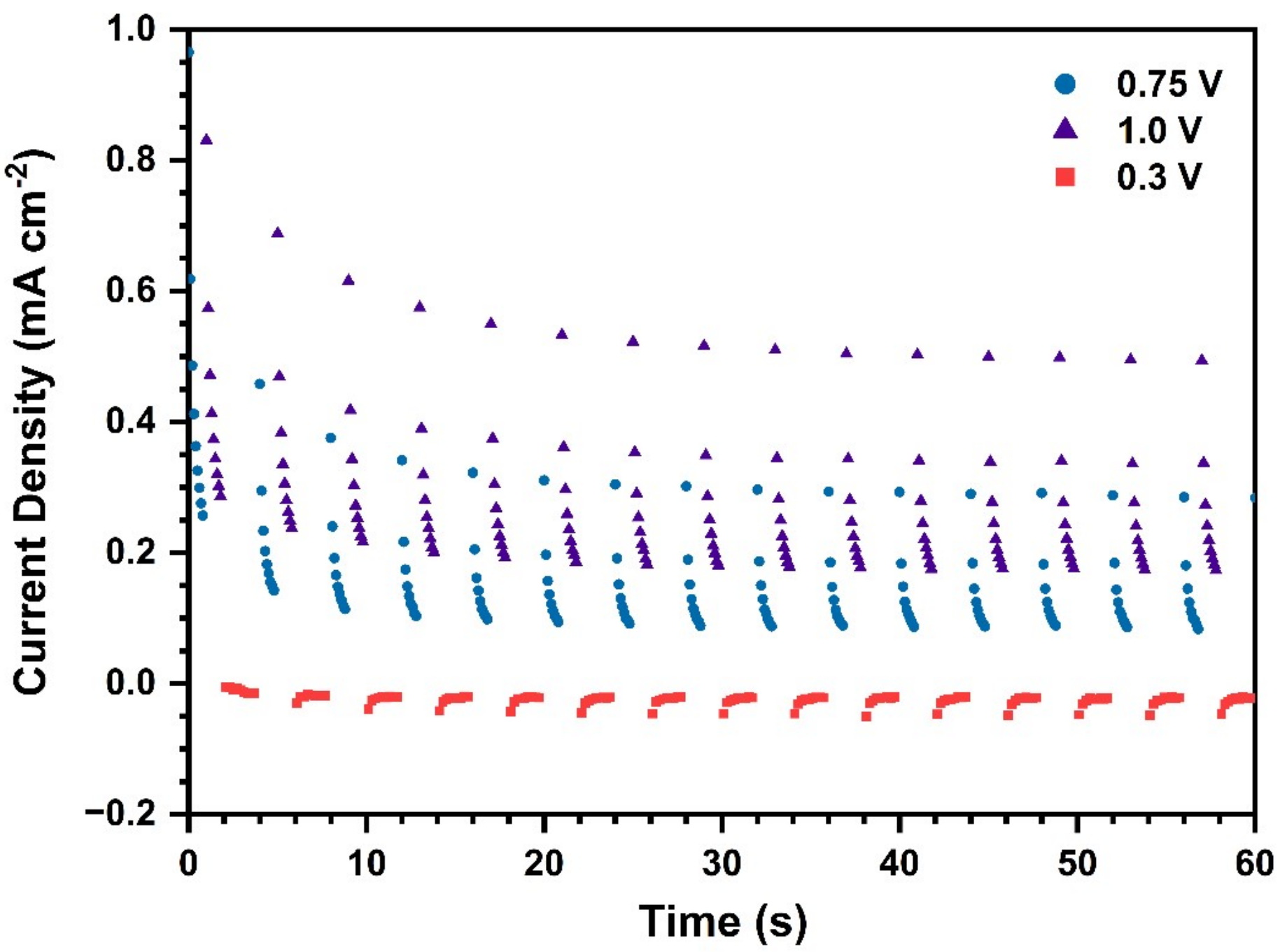

Fig. S24 Current density responses obtained using the three-step pulsed potential protocol P15 ($t_{0.75V}$ = 1 s, $t_{1.0V}$ = 1 s, $t_{0.75V}$ = 2 s) in 0.01 M glyceraldehyde and 0.1 M NaOH at 60 °C under static conditions (0 rpm).

Chemical stability test for glyceraldehyde

We performed an additional control experiment to examine the chemical stability of glyceraldehyde under the same conditions used for glycerol oxidation (0.1 M NaOH, 60 °C), but without applying an electrochemical potential. After adding glyceraldehyde to the pre-heated electrolyte, aliquots were collected at different reaction times and analyzed by HPLC (Fig. S25). At the initial stage, the glyceraldehyde peak overlapped with those of lactate and glycolate, making accurate quantification of the glyceraldehyde concentration difficult. As the reaction proceeded, the intensity of the overlapping peak progressively decreased, while the lactate peak became increasingly well resolved. After 60 min, the chromatogram showed a distinct lactate peak with little contribution from glyceraldehyde. These results demonstrate that glyceraldehyde is sufficiently stable to persist in solution after desorption from the electrode surface, while simultaneously undergoing spontaneous chemical conversion to lactate under the alkaline conditions employed in this work. This observation supports the proposed mechanism in which glyceraldehyde is released into solution and subsequently converted to lactate through a homogeneous chemical reaction.

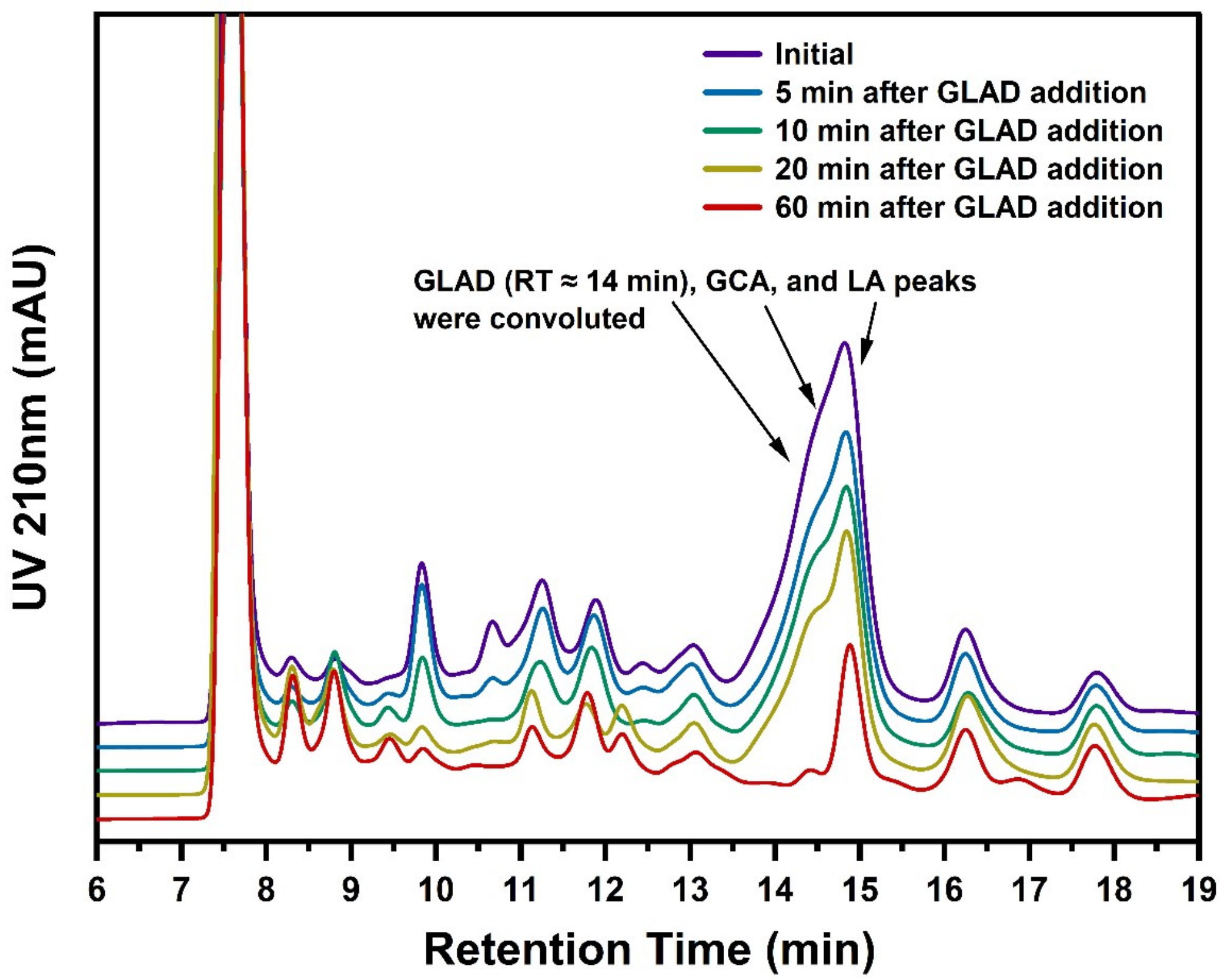

Fig. S25 Comparison of HPLC chromatograms recorded at 210 nm showing the chemical decomposition of glyceraldehyde (GLAD) in 0.1 M NaOH solution at 60 °C in the absence of electrolysis. A concentrated GLAD solution was added to pre-heated 0.1 M NaOH to obtain a final concentration of 0.01 M, followed by rapid stirring at 1000 rpm for 30 s. The solution was then maintained under static conditions and collected at different resting times before being acidified with H_2SO_4 for HPLC analysis. The peak at approximately 7.2 min corresponds to dissolved Na_2SO_4 generated during dilution and acidification. The GLAD, glycolate (GCA) and lactate (LA) peaks exhibited significant overlap and could not be fully resolved.

Supplementary References

60. T. H. M. Housmans, A. H. Wonders and M. T. M. Koper, The *J. Phys. Chem. B*, 2006, **110**, 10021-10031.

61. E. Zamburlini, K. D. Jensen, I. E. L. Stephens, I. Chorkendorff and M. Escudero-Escribano, *Electrochim. Acta*, 2017, **247**, 708-721.

62. M. Eisenberg, C. W. Tobias and C. R. Wilke, *J. Electrochem. Soc.*, 1954, **101**, 306.

63. M. Eisenberg, C. W. Tobias and C. R. Wilke, *Chem. Eng. Progr. Symp. Ser.,* 1955, **51**, 1.

64. G. D'Errico, O. Ortona, F. Capuano and V. Vitagliano, *J. Chem. Eng. Data*, 2004, **49**, 1665-1670.

65. M. H. Rausch, A. Heller and A. P. Fröba, *J. Chem. Eng. Data*, 2017, **62**, 4364-4370.

66. J. A. Trejo González, M. P. Longinotti and H. R. Corti, *J. Chem. Eng. Data*, 2011, **56**, 1397-1406.
